# Peroxisome function relies on organelle-associated mRNA translation

**DOI:** 10.1126/sciadv.abk2141

**Published:** 2022-01-12

**Authors:** Noa Dahan, Yury S. Bykov, Elizabeth A. Boydston, Amir Fadel, Zohar Gazi, Hodaya Hochberg-Laufer, James Martenson, Vlad Denic, Yaron Shav-Tal, Jonathan S. Weissman, Naama Aviram, Einat Zalckvar, Maya Schuldiner

**Affiliations:** 1 Department of Molecular Genetics, Weizmann Institute of Science, Rehovot 7610001, Israel.; 2 Department of Cellular and Molecular Pharmacology, Howard Hughes Medical Institute, California Institute for Quantitative Biosciences, Center for RNA Systems Biology, University of California, San Francisco, San Francisco, CA 94158, USA.; 3 The Mina and Everard Goodman Faculty of Life Sciences, The Bar-Ilan Institute of Nanotechnology and Advanced Materials (BINA), Bar-Ilan University, Ramat Gan 5290002, Israel.; 4 Department of Molecular and Cellular Biology, Harvard University, 52 Oxford St., Cambridge, MA 02138, USA.

## Abstract

Crucial metabolic functions of peroxisomes rely on a variety of peroxisomal membrane proteins (PMPs). While mRNA transcripts of PMPs were shown to be colocalized with peroxisomes, the process by which PMPs efficiently couple translation with targeting to the peroxisomal membrane remained elusive. Here, we combine quantitative electron microscopy with proximity-specific ribosome profiling and reveal that translation of specific PMPs occurs on the surface of peroxisomes in the yeast *Saccharomyces cerevisiae*. This places peroxisomes alongside chloroplasts, mitochondria, and the endoplasmic reticulum as organelles that use localized translation for ensuring correct insertion of hydrophobic proteins into their membranes. Moreover, the correct targeting of these transcripts to peroxisomes is crucial for peroxisomal and cellular function, emphasizing the importance of localized translation for cellular physiology.

## Teaser

Localized translation of peroxisomal membrane proteins is crucial for cellular functions.

## INTRODUCTION

Peroxisomes are central metabolic organelles whose maturation and function depends on efficient and accurate targeting of peroxisomal membrane proteins (PMPs). Correct targeting of PMPs enables completion of peroxisome maturation and function, import of peroxisomal luminal (matrix) proteins, and transfer of metabolites (*1*–*3*). Despite their crucial roles, the targeting routes for the majority of PMPs are unknown. Mutations in many of these PMPs result in severe rare disorders (*4*). Moreover, it is recently becoming evident that suboptimal peroxisomal function is not restricted to rare genetic events but correlates with aging and underlies a broad spectrum of pathological conditions from viral infectivity through cancer (*5*).

The current paradigm suggests that PMPs are targeted to peroxisomes by either of two paths. One, an indirect pathway, begins with PMPs being targeted to the endoplasmic reticulum (ER) from which they exit on pre-peroxisomal vesicles (PPVs) that later mature to, or fuse with, functional peroxisomes. The second, a direct path, involves PMP synthesis in the cytosol followed by posttranslational targeting to the peroxisome (*6*). In both of these scenarios, the accepted model is that PMP targeting depends on the essential biogenesis factor Pex19.

Pex19 is a cytosolic chaperone that binds several PMPs (in yeast: Pex3, Pex10, Pex11, Pex13, and Pex22 have all been shown to rely on Pex19 for their targeting) and escorts them to their final destination (*7*–*10*). For many years, the function of Pex19 was thought to explain the targeting of all PMPs. However, multiple lines of evidence are now accumulating to suggest that PMP targeting is more complicated and intricate than previously thought. First, although a Pex19 binding motif was found (*7*), many membrane proteins do not have it, nor have they been shown to bind Pex19; second, some PMPs, also of those that bind Pex19, can be targeted to peroxisomes or PPVs independently of Pex19 (*11*); third, Pex19 also targets a subset of mitochondria and lipid droplet membrane proteins (*12*, *13*), implicating it as a multifunctional chaperone. Therefore, we reasoned that particular PMPs may be targeted to the peroxisomal membrane by a Pex19-independent alternative route.

Conceptually, membrane proteins translated freely in the cytosol and targeted only posttranslationally increase the danger of being mistargeted or aggregated, both bearing toxic effects on cells (*14*, *15*). Therefore, it stands to reason that such highly hydrophobic proteins will depend on organelle-associated translation (which we here will also term localized translation). In such events, particular mRNA transcripts are translated proximal to the target membranes of their resulting proteins, thus averting their requirement to travel through the cytosol to target correctly. Studies aimed to reveal the extent and mechanisms of localized translation have focused mostly on the ER where translation is often coupled with translocation through the ER membrane (*16*, *17*) as well as on mitochondria and chloroplasts (*18*–*21*). As for peroxisomes, this phenomenon remained unstudied.

The possibility that peroxisomal protein targeting can occur by localized translation was raised to explain the observations that the mRNAs of several PMPs were visualized as colocalizing with peroxisomes using an in vivo mRNA tagging approach (MS2 loops, *MS2L*) (*22*). Special attention was given to Pex14, an abundant PMP that is a crucial component of the peroxisome import machinery and is essential for peroxisome biogenesis (*23*, *24*). A high percentage of *PEX14-MS2L* mRNA transcripts were shown to colocalize with peroxisomes; however, their localization was shown to be partly dependent on Pex14 translation (*22*). Because Pex14 contains a Pex19 binding domain at its N terminus (*7*), the most trivial explanation would be that Pex19 binds the nascent Pex14 and targets it to peroxisomes during translation, bringing the entire translating ribosome and mRNA with it. In this scenario, the mRNA localization to peroxisomes would simply be a by-product of Pex19-mediated targeting. Hence, the observation of localized mRNA transcripts did not provide an answer to the long-standing question—Is there a Pex19-independent pathway for PMPs to be correctly targeted to the peroxisomal membrane?

## RESULTS AND DISCUSSION

**Fig. 1.**
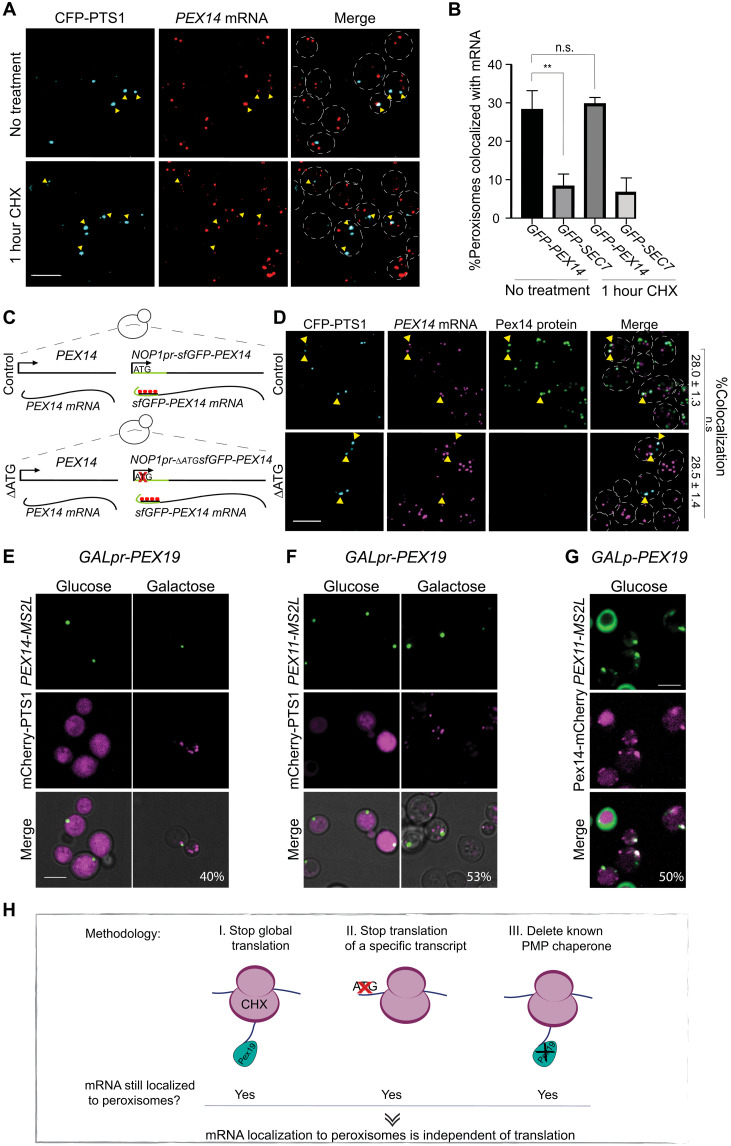
Peroxisomal mRNA localization is not dependent on translation or Pex19. (**A**) Translation inhibition reveals that colocalization of *PEX14* mRNA and peroxisomes is translation independent. The maximum intensity projections of confocal images of smRNA-FISH assay of *sfGFP*-*PEX14* (TAMRA, red) following 1-hour incubation with CHX are shown. Peroxisomes in cyan (CFP-PTS1); yellow arrowheads highlight colocalization between *PEX14* mRNA and peroxisomes; yeast periphery in dashed white line. (**B**) Quantification of (A). Random colocalization threshold calculated by mRNA of Golgi *SEC7* (*n* = 150; **P < 0.05, unpaired *t* test). n.s., not significant. (**C**) Schematic description of interference with *PEX14* translation. *sfGFP*-*PEX14* under the *NOP1* promoter (*NOP1pr*) was integrated into the HO (homothallic switching endonuclease) locus locus either with the start methionine (+ATG) or without (−ATG). (**D**) Representative smFISH micrographs of +ATG and −ATG strains described above. Mature peroxisomes in cyan (CFP-PTS1), *PEX14* mRNA in magenta (TAMRA), and Pex14 protein in green (GFP); yellow arrowheads indicate colocalization. Percentage of peroxisomes colocalizing with *sfGFP-PEX14* mRNA (*n* = 50; *P* > 0.05, unpaired *t* test). (**E**) Representative micrograph from time-lapse experiments of strains following induction of *GALpr*-*PEX19*. *PEX14-MS2L* in green (MS binding coat protein–GFP) and peroxisomes in magenta (mCherry-PTS1). Percentage of mRNA granules colocalizing with peroxisomes. *n* = 100. (**F**) Same as (E) but visualizing *PEX11-MS2L* mRNA (green). (**G**) Micrograph showing *PEX11-*MS2L mRNA (green) colocalized with pre-peroxisomal vesicle (PPV) in magenta (Pex14-mCherry) in the absence of Pex19 (*GALpr-PEX19* grown on glucose). Percentage of mRNA granules colocalizing with PPVs. Scale bar, 5 μm. (**H**) Graphical summary of Fig. 1—three experimental methodologies suggest that mRNA localization to peroxisomes is independent of translation.

We set out to answer this question in the model organism, the yeast *Saccharomyces cerevisiae*, that has been shown to have the same peroxisomal targeting components as are found in human cells (*25*). We decided to first focus on Pex14 because its mRNA was shown to be localized to peroxisomes and because it can be targeted to PPVs even in the absence of Pex19 (*22*, *26*). If Pex14 is targeted by Pex19 alone and this is the reason that its mRNA accumulates in proximity to peroxisomes, then mRNA localization to peroxisomes should be dependent on translation in general—specifically, translation of *PEX14* mRNA—and on the presence of Pex19. To rigorously test this hypothesis, we performed three complementary experiments. First, we arrested global cellular translation by adding the translation inhibitor cycloheximide (CHX) and assayed the effects on colocalization of *PEX14* transcripts with peroxisomes. To avoid adding long stretches of sequence to the 3′ untranslated region (UTR), as is done in the *MS2L* method, we detected superfolder green fluorescent protein (sfGFP)–tagged *PEX14* mRNA by single-molecule RNA fluorescence in situ hybridization (smRNA-FISH) (*27*). We verified that the CHX treatment blocked translation by the decrease of sfGFP-Pex14 expression levels (fig. S1). However, despite the reduction in translation, the percentage of peroxisomes that colocalized with *sfGFP-PEX14* mRNA before and after CHX treatment remained the same (~30%) and higher than the negative control (Fig. 1, A and B). Hence, colocalization of *PEX14* mRNA with peroxisomes was independent of global translation. Next, we prevented the translation of *sfGFP-PEX14* by deleting its initiating methionine (∆ATG) on a single, genomically expressed, copy in cells expressing also the native *PEX14* gene (to ensure that functional peroxisomes still exist). Although sfGFP-Pex14 was not expressed in the absence of its ATG (fig. S2), here, too, we observed no reduction in colocalization of *sfGFP*-*PEX14* mRNA with peroxisomes (Fig. 1, C and D). This supported our proposition that the localization of *PEX14* transcripts to peroxisomes is independent of protein translation. Third, to determine whether the chaperone Pex19 has a role in the peroxisomal localization of *PEX14* mRNA, we expressed *PEX19* under the galactose inducible/glucose repressible promoter (*GALpr*) because complete deletion of Pex19 results in no peroxisomes and inability to track any localization events. As this experiment requires tracking a dynamic process, we visualized the localization of *PEX14* mRNA by the MS2L system that allows in vivo imaging of mRNA granules (*28*). To verify our results, we also performed this experiment on an additional PMP transcript that was shown to colocalize with peroxisomes, *PEX11-MS2L* (*22*). As expected for both, when Pex19 expression was restored by growth in galactose and mature peroxisomes started to appear, the mRNA granules colocalized with them. Even when Pex19 was fully repressed by growth in glucose, and Pex19 absence was verified by inability of mature peroxisomes to form (fig. S4), we could still detect mRNA granules (Fig. 1, E and F). Moreover, time-lapse experiments showed that these granules mark sites of subsequent formation of mature peroxisomes (fig. S3) and are fully associated with PPVs (Fig. 1G), known to exist in the absence of Pex19 (*10*).

The results of all three experiments demonstrate that localization of PMP transcripts can occur independently of translation and Pex19 (Fig. 1H). Moreover, they highlight the possibility that mRNA positioning to peroxisomes marks an early step in peroxisome biogenesis.

**Fig. 2.**
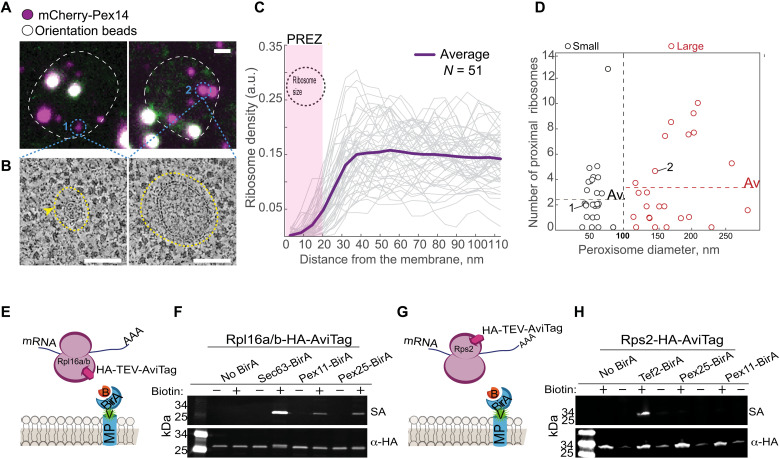
A subset of ribosomes is proximal to the peroxisomal membrane despite a REZ. (**A**) Fluorescent micrographs of yeast. Peroxisomes (small-1 or large-2) in magenta (mCherry-Pex14). TetraSpeck beads (white) used for correlation. Scale bar, 1 μm. (**B**) Electron tomograms of peroxisomes from (A). Surrounding each peroxisome is a peroxisomal ribosome exclusion zone (PREZ; yellow dashed line). Yellow arrowhead shows ribosomes adjacent to the membrane. Scale bar, 100 nm. (**C**) Graph of ribosome density (each gray line = one peroxisome) as a function of distance from the membrane. The PREZ (yellow dashed line) is approximately the ribosome size (black circle). *n* = 51. a.u., arbitrary units. (**D**) Modeled peroxisome size distributions separate small (~50 nm diameter) and large (>100 nm diameter) peroxisomes. Dashed line is the average number of proximal (>20 nm from membrane) ribosomes in each group. “1” and “2” are peroxisomes from (A). *n* = 51. (**E**) Schematic representation of the proximity labeling assay. An abundant membrane protein (MP) fused to mVenus (v, green) and BirA (blue crescent) in cells that express Rpl16a/b fused to HA followed by a Tobacco Etch Virus (TEV) protease–cleavable AviTag. The AviTag is biotinylated upon addition of biotin (B, orange) only in ribosomes that are proximal (less than 10 nm) to the BirA-fused MP. (**F**) Western blot analysis of cells described above on the background of either Sec63 (ER-MP, positive control), Pex11, or Pex25 (abundant PMPs) fused to mVenus-BirA. Biotinylation detected by fluorescent streptavidin (SA; green). α-HA antibody used as loading control (red). (**G**) A schematic representation of (H) that is similar to (E), but HA-TEV-AviTag is fused to Rps2. (**H**) Western blot analysis as in (F) but with Rps2-AviTag. Cytosolic protein Tef2 is a positive control.

Colocalization of the mRNAs of *PEX11* and *PEX14* with PPVs suggests a role for peroxisomal-localized translation in growth and maturation of peroxisomes. We therefore hypothesized that PMP transcripts are translated in proximity to PPVs or the mature peroxisomal membrane. For onsite translation to occur, ribosomes must be associated with the peroxisomal membranes. Because such an association has not been previously reported, we first visualized yeast peroxisomes at high resolution to assay their association with ribosomes. To enable accurate recognition of these tiny organelles (diameter of 0.1 to 1 μm), we applied correlative light and electron microscopy (*29*, *30*). We correlated the fluorescent signal of mCherry-Pex14 to identify 51 peroxisomes that varied in their diameter. For each peroxisome, we acquired high-resolution electron tomography data, modeled their membranes, and determined positions of neighboring ribosomes (Fig. 2, A and B, and movie S1). To our surprise, all peroxisomes were surrounded by a 20-nm region that was mostly devoid of ribosomes (Fig. 2, B and C). Such ribosomal exclusion zones (REZs) were previously described around endocytic invaginations that are surrounded by an actin mesh (*31*).

The absence of ribosomes in the peroxisomal REZ (which we named PREZ) emphasized a number of ribosomes (one to three ribosomes per organelle) that crossed it and were positioned adjacent to each of the peroxisomal membranes that we modeled. The average number of peroxisomal membrane–associated ribosomes (closer than 20 nm) did not correlate with peroxisome size. There were roughly the same number of proximal ribosomes on small and large peroxisomes (Fig. 2D). This suggests that the PREZ-crossing ribosomes are not randomly found in proximity to the peroxisomal membrane.

To support the observation that ribosomes can be found in proximity to peroxisome membranes, we modified the previously used ribosome proximity labeling assay (*32*) to detect ribosomes proximal to peroxisomes. We spatially restricted the expression of a biotin ligase (BirA) to peroxisomes by fusing it to either Pex25 or Pex11, both abundant PMPs (fig. S5, A and B). In these same strains, the large subunit ribosomal protein, Rpl16a/b, was fused to an AviTag, which acts as a specific biotin acceptor peptide (Fig. 2E). Following a biotin pulse, we detected proximal ribosomes by streptavidin in both strains, supporting our observation that ribosomes reach a distance of under 10 nm from the peroxisomal membrane (Fig. 2F). This biotinylation did not take place when the AviTag was fused to a small ribosomal subunit protein 2 (RPS2) (Fig. 2, G and H). Because the nascent chain emerges from the large subunit and because orientation-dependent association is also occurring at the ER (*32*), we conclude that ribosomes are found in proximity to peroxisomes, in a controlled and oriented manner, potentially poised for translation.

**Fig. 3.**
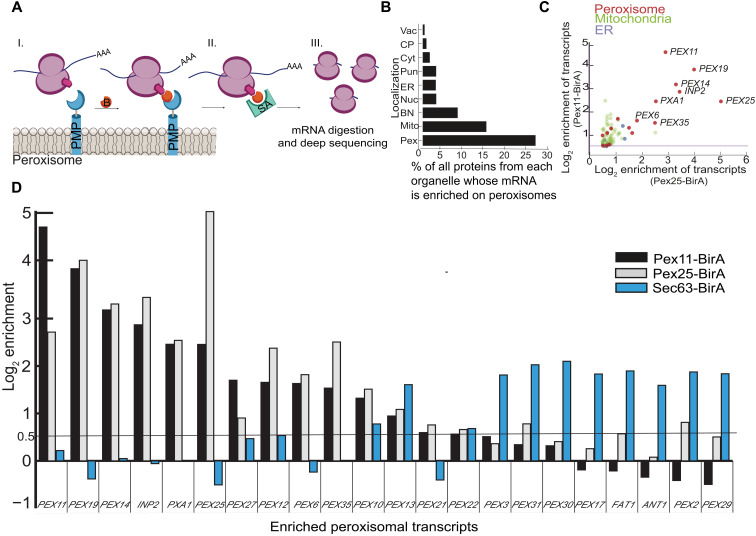
Peroxisomal ribosomes translate a specific subset of PMPs. (**A**) Schematic representation of mRNA profiling following proximity labeling: (I) Pex25/Pex11 was fused to mVenus-BirA, biotinylating proximal ribosomes (Rpl16a/b) upon addition of biotin (B, orange). (II) Biotinylated ribosomes were purified using streptavidin beads. (III) Protected mRNA was subjected to sequencing. (**B**) Percent translational enrichment per cellular local of the total proteome. Cellular localization of proteins tagged at the N terminus (*50*). Vac, vacuole; CP, cell periphery; Cyt, cytosol; Pun, punctate; ER, endoplasmic reticulum; Nuc, nucleus; BN, bud neck; Mito, mitochondria; Pex, peroxisomes. (**C**) Relative enrichment of mRNAs in the ribosome profiling, color-coded based on protein cellular localization. (**D**) Relative enrichment levels of PMP transcripts from either peroxisome (Pex11-BirA, black; Pex25-BirA, gray) or specific ribosome profiling (Sec63-BirA in blue) (*33*). Enrichment cutoff in black line (log_2_ enrichment = 0.5). A transcript was considered enriched when it was significant in both Pex11- and Pex25-BirA experiments. Clear subsets of proteins can be seen, which are translated either in proximity to peroxisomes or in proximity to the ER.

To uncover whether the peroxisomal-proximal ribosomes translate specific transcripts, we performed peroxisome-specific ribosome profiling (*21*, *32*). To do this, we performed a streptavidin pulldown of the ribosomes biotinylated by the peroxisome-localized BirA (Pex-BirA). Because this procedure is done after translation is halted by CHX, we first made sure that the colocalization levels of mRNA transcripts with peroxisomes are not affected by CHX addition (Fig. 3A and fig. S6). We then sequenced protected mRNA segments to uncover 40 transcripts that were enriched in the Pex-BirA–tagged strains relative to a total ribosomal sample that was not subject to a streptavidin pulldown (table S1). Of these, peroxisomal transcripts were highly enriched, specifically those of PMPs including *PEX11*, *PEX14*, *INP2*, *PXA1*, *PEX25*, *PEX27*, *PEX12*, *PEX35*, *PEX10*, *PEX13*, *PEX21*, and *PEX22.* We also observed translation of *PEX6* mRNA whose protein is only peripherally associated with the membrane and, unexpectedly, transcripts of *PEX19* chaperone itself (Fig. 3B and fig. S7, A to C). We validated the peroxisomal localization of several of these transcripts using smRNA-FISH (fig. S8). The fact that most of the highly enriched transcripts of peroxisome proteins were PMPs suggests that peroxisome-localized translation is mostly used by hydrophobic proteins that could potentially aggregate in the cytosol or be mistargeted to other membrane destinations. The enrichment of *PEX19* mRNA may suggest localized translation as a means to concentrate Pex19 in the vicinity of peroxisomes.

It was previously demonstrated by ER-specific ribosome profiling experiments that the transcripts of some PMPs are translated in vicinity to the ER before the targeting of the mature proteins to peroxisomes (specifically *PEX31*, *PEX17*, *FAT1*, *PEX29*, *ANT1*, *PEX2*, and *PEX3*) (*32*, *33*). Low colocalization of these mRNAs to peroxisomes was supported by studies using the MS2L system (*22*). To see how the transcripts of PMPs are distributed between the two organelles during their translation, we compared the enriched transcripts between peroxisomal and ER-associated ribosome profiling data. The comparison revealed the existence of two PMP subsets, one that is locally synthesized by ER-associated ribosomes and the other by peroxisome-associated ones (Fig. 3D).

To assay whether the mRNAs of locally translated transcripts arrive via mRNA binding proteins, we tried to computationally uncover shared sequences or known recognition motifs for RNA binding proteins in our peroxisomal ribosome profiling–enriched transcripts, but without success. However, targeting of these transcripts may rely on a combination of their primary, secondary, and tertiary structures that enable recognition by trans-elements involved in mRNA localization (*34*). Alternatively, a cotranslational targeting machinery, akin to the signal recognition particle, may be present and not yet identified. The machinery allowing this localized translation must therefore still be explored.

**Fig. 4.**
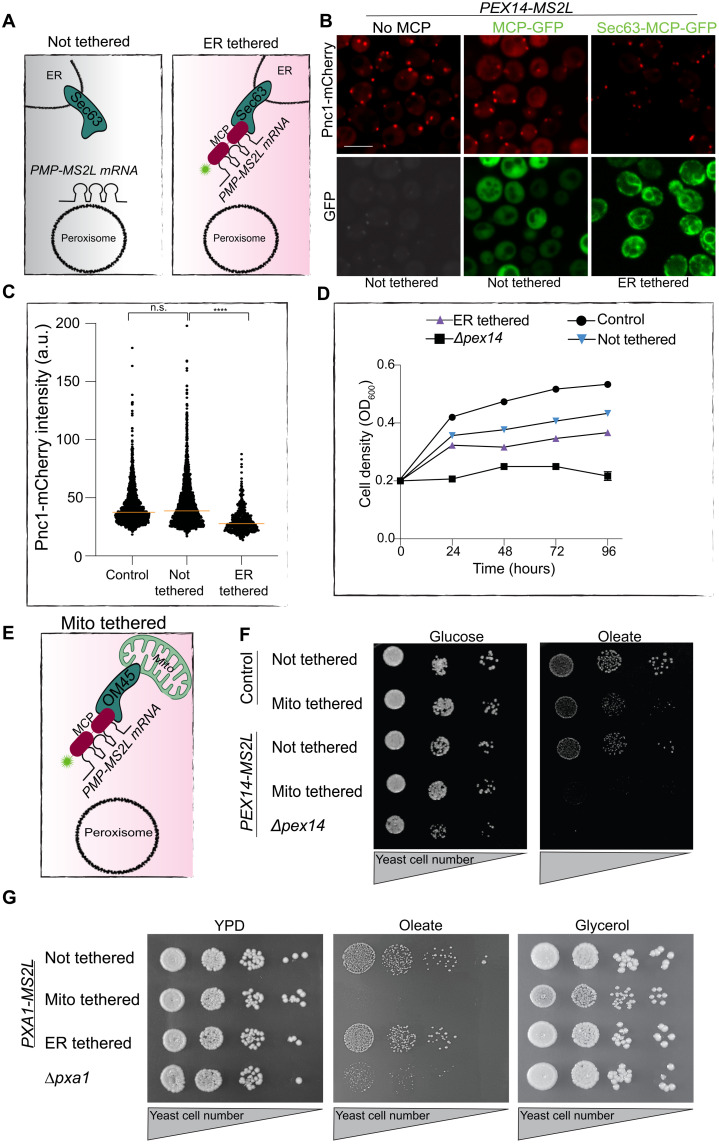
Mislocalization of peroxisomal mRNAs impairs peroxisomal and cellular function. (**A**) Illustration of how an mRNA-MS2L is tethered to the ER using Sec63 fused to MCPx2-GFP. (**B**) Representative micrograph of fluorescence intensity of peroxisomal matrix Pnc1-mCherry (red) when *PEX14-*MS2L mRNA was either not tethered (left) bound by cytosolic MCPx2-GFP (green, middle) or ER-tethered (green, right) during growth in glucose (*22*). (**C**) Scatter plot of fluorescent intensity of 876 Pnc1-mCherry puncta in strains from (B). Median, orange line (significance determined by Mann-Whitney unpaired *t* test) (**D**) Growth curve of indicated strains showing that ER tethering of *PEX14*-*MS2L* mRNA slows yeast growth on oleate. Assay in triplicate; error bars are presented but are too small to detect. (**E**) Illustration describing the tethering of mRNA-MS2L to mitochondria as in (A) but using Om45 fused to MCPx2-GFP. (**F**) Drop dilution assay, 7 days on oleate-containing medium, reveals that mislocalization of *PEX14* to mitochondria impairs growth similarly to Δ*pex14*. (**G**) Drop dilution assay reveals that mitochondria-tethered *PXA1*-MS2L mRNA impairs strain growth in oleate and slows growth in glycerol (performed in triplicate; representative images shown).

Several of the enriched transcripts encode for PMPs that are essential for peroxisomal functions. If their proper targeting to peroxisomes is dependent on localized translation, disrupting it should lead to measurable physiological consequences. To assay this, we first focused on Pex11, which is important for peroxisome growth and division (*35*). To assay the importance of its localized translation, we tethered the endogenously tagged *PEX11-MS2L* to an alternate location, the ER, by fusing Sec63 (an ER membrane protein) to an *MS2L* binding protein, MCP (MCPx2-GFP) (Fig. 4A). The ER-localized Sec63-MCPx2-GFP anchored the MS2L-tagged mRNA onto the ER periphery (fig. S9), as was previously shown (*36*). Mislocalizing *PEX11* mRNA to the ER resulted in fewer peroxisomes, suggesting that mRNA localization plays a key role in proper translation and protein targeting/translocation for this PMP (fig. S10). We next tested Pex14, which is essential for peroxisome biogenesis. When we tethered *PEX14-MS2L* to the ER using the above approach, we detected (Fig. 4B) and quantified (Fig. 4C) a significant reduction in the intensity of the peroxisomal marker Pnc1-mCherry (*37*). To assay whether this decreased intensity is due to incomplete maturation of peroxisomes, we measured growth in conditions where peroxisomes become essential for yeast cell survival, oleate as the sole carbon source. The mistargeted *PEX14* transcript reduced cell viability in this condition (Fig. 4D). To exacerbate the phenotype, we tethered *PEX14-MS2L* to mitochondria, which are not as closely associated to peroxisomes as the ER (*38*). Tethering to mitochondria was performed by endogenously fusing the outer mitochondrial membrane protein OM45 with MCPx2-GFP (Fig. 4E and fig. S11). This demonstrated that cells with mitochondrial tethered *PEX14* mRNA could not grow on oleate at all, although they grew normally on glucose-supplemented plates (Fig. 4F). The extent of the phenotype was similar to a complete deletion of *PEX14* gene, highlighting the inability of the nascent Pex14 protein to reach the peroxisomal membrane if not synthesized in its vicinity, despite normal Pex19 presence in these cells. We verified that the effect is not due to *PEX14* mRNA being toxic to mitochondria, as the cells in which *PEX14* mRNA was tethered to mitochondria grew similarly to control cells on plates requiring mitochondrial respiration (glycerol as a sole carbon source) (fig. S12).

Last, we applied a similar assay to *PXA1-MS2L*. Pxa1 is a subunit of the peroxisomal adenosine 5′-triphosphate (ATP)–binding cassette (ABC) transporter complex that is essential for oleate uptake into peroxisomes and is a homolog of the human adrenoleukodystrophy transporter (ALDp), mutations in which cause X-linked adrenoleukodystrophy (*39*, *40*). When *PXA1* mRNA was tethered to mitochondria, cells could not grow on oleate as a sole carbon source and also exhibited a small colony growth phenotype and reduced growth kinetics on glycerol as a sole carbon source (Fig. 4G and fig. S13). Because *pxa1*-deleted yeast properly grow on glycerol as a sole carbon source (Fig. 4G and fig. S13), the phenotype cannot be due to loss of Pxa1 activity, suggesting that toxicity arises from integration of Pxa1 into the mitochondrial membranes when its mRNA is tightly tethered at this local. Such a phenomenon was described before, when transcripts that are locally translated on the ER were mistargeted to mitochondria and caused mitochondrial defects (*15*).

Our work shows that yeast use localized translation on peroxisomal membranes as a way to ensure accurate and efficient membrane protein targeting to this important organelle. We show that targeting of the localized mRNAs is independent of the chaperone Pex19, suggesting that a parallel mechanism to the one previously described exists for PMP targeting. Our work suggests that mistargeting of peroxisomal transcripts not only interferes with peroxisomal functions but also burdens other organelles. It could be that the main advantage of localized translation is to enable targeting specificity, a function that Pex19, which also targets membrane proteins to other organelles, cannot fully perform (*13*, *14*).

Our work puts forward a paradigm for how targeting specificity could arise by localized translation of specific transcripts, encoding hydrophobic proteins, proximal to peroxisomal membranes. Furthermore, while a clear association between organelle malfunction disorders and mislocalization of transcripts has not yet been established, our results suggest that such a connection may exist. Our findings will undoubtedly provide ample ground for such investigations.

More globally, the cell biology of localized translation has, until now, focused on the ER, mitochondria, and chloroplasts. Our findings put peroxisomes as a new destination for localized translation and suggest that maybe more such destinations exist, previously overlooked due to the sophisticated tools required to uncover their presence.

## MATERIALS AND METHODS

### Yeast growth medium

Synthetic medium used in this study contains yeast nitrogen base (6.7 g/liter) with ammonium sulfate (Conda Pronadisa #1545) and either 2% glucose (SD), 3% glycerol (SGly), 2% galactose (SGal), or 0.2% oleic acid (Sigma-Aldrich) + 0.1% Tween 80 (SOle), with complete amino acid mix (optimized minimal media composition) (*41*), unless written otherwise. When geneticin antibiotics are used, the medium contains yeast nitrogen base (0.17 g/liter) without ammonium sulfate (Conda Pronadisa #1553) and monosodium glutamic acid (1 g/liter) (Sigma-Aldrich #G1626) instead of yeast nitrogen base with ammonium sulfate. When mentioned, geneticin (500 mg/liter; G418) (for medium) and nourseothricin (200 mg/liter) (WERNER BioAgents “clonNAT”) were used.

### Yeast growth assay

All growth assays were performed in triplicate. Cells were grown to stationary phase in SD and proper selections. Cells were then diluted to 0.2 optical density (OD) for overnight growth in 0.1% glucose followed by a second dilution to 0.2 OD in SOle. For Fig. 4D, OD was measured manually every 24 hours following two washes in double distilled water, and the growth rate of each strain was normalized to its control strain that expresses only the selection cassette without any further modification. The growth assay in fig. S13 was done in a 96-well plate reader for automatic OD measurements.

### Yeast drop assay

Cells were grown to stationary phase in liquid medium followed by dilution to 0.2 OD in 0.1% glucose-supplemented medium. When cells reached 0.4 to 0.6 OD, 1 OD was collected and washed with DDW. Five 1:10 serial dilutions were performed in medium. All dilutions were plated on SD or SOle as well as on YPD (yeast extract, peptone, and dextrose) or YP + 3% glycerol agar plates. Colonies that were grown on YPD and SD agar plates were imaged 2 to 3 days after plating. YPGly-grown colonies were imaged 4 to 5 days after plating, and YPOle-grown colonies were imaged 7 to 10 days after plating.

### Transformations and genetic manipulations

Cells were genetically manipulated using a transformation method that includes the usage of lithium acetate, polyethylene glycol, and single-stranded DNA (*42*). All primers for manipulations and validation were designed using Primers-4-Yeast (*43*).

### Single-molecule RNA fluorescence in situ hybridization

The protocol used for smRNA-FISH was slightly modified from (*44*). Cells were grown from 0.4 to 0.6 OD in SD medium with proper selections; 3 ODs in total were fixed with 4% formaldehyde (Sigma-Aldrich), spheroplasted with lyticase for 30 min at 30°C, and washed carefully.

#### Probe hybridization

Spheroplasted cells were hybridized with DNA probes overnight at 30°C. For the smRNA-FISH experiments described in Fig. 1 (A and D) and in figs. S5 and S7, a set of 40 probes that was designed against sfGFP was used. The probes were designed by the online program Stellaris RNA Probe Designer from Biosearch Technologies (Novato, CA, USA) and ordered conjugated to the TAMRA fluorescent dye. The probes used for smRNA-FISH experiments in fig. S8 are a mixture of three different DNA oligos matching the MS2L sequence that were manually conjugated to Cy3 as described in (*45*) (a gift of J. Gerst).

### Imaging and analysis

#### For smRNA-FISH

Cells were washed from the probe with formamide (Sigma-Aldrich)–based wash buffer and placed on a 384-well glass-bottom microscope plate (Matrical Bioscience) coated with concanavalin A. After 20 min, cells were washed with wash buffer to remove nonadherent cells and to obtain a cell monolayer. The plate was then imaged in an automated inverted fluorescent microscope system (Olympus) harboring a spinning disc module using a 60× oil lens (numerical aperture, 1.42) with a Hamamatsu ORCA-Flash 4.0 camera. Images were recorded with the cellSens dimension software (Olympus) in four channels: GFP (excitation wavelength, 488 nm), mCherry (excitation wavelength, 561 nm), 4′,6-diamidino-2-phenylindole (DAPI) (excitation wavelength, 405 nm), and brightfield. In each position, three-channel Z-stack images were taken with a step size of 350 nm for a total of 3.5 μm. Each Z-plane image was of size 2048 × 2048 pixels. Images were analyzed in Fiji image processing package (*46*) used for Z-stack conversion to a single-plane image based on maximal projection, and the cell counter tool was used to manually count areas of colocalization.

#### For peroxisomes intensity analysis

Cells were grown with proper selections and plated on 384-well glass-bottom plates. Following single-plane imaging as above, cells were segmented on the basis of their GFP background. Then, 876 Pnc1-mCherry puncta representing peroxisomes were selected randomly for segmentation by the scanR acquisition software and measured for their intensity.

### In vivo visualization of peroxisomal mRNA

The mRNA of *PEX11* and *PEX14* in the absence or presence of Pex19 was visualized in strains that were previously described in which these peroxins are tagged with MS2L (version 3). Strains were a gift from J. Gerst. These same strains were further manipulated to replace the native promoter (*NATIVEpr*) of *PEX19* with a *GALpr* and transformed with a 2u expression plasmid bearing the site-specific RNA binding coat protein of the bacteriophage MS2 (MCP) fused to three repeats of GFP. Cells were grown to stationary phase on a 96-well plate in SD or SGal without histidine. Cells were then regrown to mid-log phase in SOle medium for 5 hours. For the time-lapse experiment presented in fig. S3, to follow the formation of peroxisomes upon *GALpr*-*PEX19* expression induction, cells were incubated overnight with SD, followed by SOle incubation for 5 hours, and then SGal-supplemented medium was added before overnight visualization.

#### Visualization

In vivo imaging of yeast cells was performed using the VisiScope Confocal Cell Explorer system, composed of a Zeiss Yokogawa spinning disk scanning unit (CSU-W1) coupled with an inverted Olympus microscope (IX83; 60× oil objective). To visualize mRNA granules, we applied the excitation wavelength of 488 nm for GFP for 500 ms, and to visualize mCherry, we used the 561-nm excitation channel for 1500 ms. Images were taken by a connected pco.edge scientific complementary metal-oxide semiconductor (sCMOS) camera controlled by VisView software. For the time point experiment presented in fig. S3, an image was acquired every 5 min starting from the time the cells were moved into galactose medium during 10 hours.

### Correlative microscopy and measurements of ribosome proximity

An overnight starter of yeast cells expressing *TEF2pr-mCherry-PEX14* was grown in YPD to stationary followed by back-dilution to reach mid-logarithmic growth phase. Twelve ODs of cells were harvested, pelleted, and subjected to high-pressure freezing.

#### High-pressure freezing

Freeze substitution and resin embedding were done exactly as described earlier (*47*). The embedded samples were sectioned to the nominal thickness of 200 nm using a Leica UC7 microtome and mounted on 200-mesh copper grids with continuous carbon support film (Electron Microscopy Sciences).

#### Fluorescence signal detection

TetraSpeck beads of 100 nm diameter (Thermo Fisher Scientific) were diluted in phosphate-buffered saline (PBS) and deposited on the sample surface to be used as correlation markers. The grids were sandwiched between two round coverslips with small amount of PBS, and Z-stacks were acquired in several locations using a VisiScope Confocal Cell Explorer system, composed of a Zeiss Yokogawa spinning disk scanning unit (CSU-W1), an inverted Olympus microscope (IX83; 100× oil objective; excitation lasers of 488 nm for GFP channel and 560 nm for mCherry channel), and a pco.edge sCMOS camera controlled by VisView software. After imaging, the grids were washed in distilled water and dried. The maximal projections resulting from Z-stacks were used to perform correlation and guide electron microscopy data acquisition.

#### Electron microscopy

Before electron microscopy, gold beads of 15 nm diameter were deposited on the sample surface as tomography fiducials and the samples were contrasted with Reynold’s lead citrate for 7 min. Electron microscopy data were collected on an FEI Tecnai G2 F20 transmission electron microscope using a Gatan UltraScan 4000 charge-coupled device detector operating at 2 × 2 binning under the control of SerialEM software. Large-area overviews used for correlation were collected as montages at a nominal magnification of ×7800 (calibrated pixel size, 2.818 nm) and large defocus (−100 μm) to visualize the TetraSpeck beads; dual-axis tomograms were collected with the angular range from 60° to −60° and 1° increment at a nominal magnification of ×19,000 (calibrated pixel size, 1.159 nm).

#### Correlation

Correlation was performed using MATLAB scripts introduced previously (*48*), and tomograms were reconstructed using IMOD package (*48*). In total, we collected and reconstructed 31 tomograms that, according to correlation, contained 66 fluorescent mCherry spots of different intensity. Of these, we excluded the spots that did not contain any spherical objects that could not be unambiguously assigned and spherical objects that contained only a small part of their volume within the cross section. Overall, we modeled 51 spherical membranes using IMOD (*47*). In three cases, there were two vesicles at the same fluorescent spot, so we modeled and included in the dataset both of them.

#### Ribosome localization

We cropped small tomographic volumes 100 pixels around each modeled membrane (in *X* and *Y*) and segmented these volumes using pixel segmentation workflow in ilastik with a label for ribosomes and a label for everything else (*49*). The segmented ribosome data were further smoothened and segmented into separate blobs using watershed transform implemented in MATLAB Image Processing Toolbox. Centroid of each blob was determined and imported back to IMOD as a model containing a set of scattered points. Then, each ribosome model was manually examined and corrected in IMOD so that each model point corresponded to one ribosome center. Typically, a model contained 200 to 400 ribosomes in a 100-pixel area around the peroxisome membrane. Ribosomes were unambiguously identified in our data as uniformly sized electron-dense bodies of around 20 nm (18 pixels) diameter. All subsequent measurements were performed using MATLAB Image Processing Toolbox. We measured the smoothened ribosome density at different distances from peroxisomal membrane to account for different diameters of peroxisomes (that greatly affects the total number of ribosomes visualized). To do this, each ribosome from the verified model was projected in a volume as a sphere of 9-pixel radius (diameter around 20 nm). The peroxisome membrane was projected in a similar volume and consequently morphologically dilated with a 5-pixel radius spherical structuring element to create 20 shell-like masks, each 5-pixel thick. These masks were applied to the volume with projected ribosomes to calculate ribosome density in each shell as a fraction of shell volume occupied by simulated ribosome pixels. The ribosome density in each shell was plotted as a function of average distance of each shell from the peroxisome membrane to create a density profile for each peroxisome, and all such profiles were averaged to determine the size of REZ—a space around peroxisomal membrane with reduced ribosomal density. To calculate the total number of ribosomes proximal to each peroxisomal membrane that was modeled, a pairwise distance between each ribosome and membrane was calculated. The ribosomes with the distance of less than 20 nm were considered proximal.

### Proximity labeling assay

The ribosomal subunits Rpl16a/b were conjugated to AviTag (biotin acceptor peptide), and Pex25 and Pex11 were conjugated to BirA (biotin ligase), allowing the specific biotinylation and streptavidin pulldown of ribosomes in close physical proximity to the peroxisome membrane. By comparing the ribosomal footprints obtained from the total ribosome fraction and the streptavidin-pulled fraction, peroxisome-localized translation enrichment was measured. Biotin induction was carried out at mid-logarithmic growth phase in the presence of CHX, which was added to the medium 2 min before the addition of biotin, at a final concentration of 100 μg/ml. Biotin pulse was carried out for either 2 or 5 min. We verified that the addition of CHX did not change the percentage of PMP mRNAs that colocalized with peroxisomes by smRNA-FISH (fig. S6). To induce biotinylation, d-biotin was added to the medium to a final concentration of 10 nM and biotinylation was allowed to proceed for 4 min at cell growth temperature. Cells were snap-frozen and kept for Western blot analysis (*21*, *32*).

### Protein extraction and Western blot

For protein extraction, pellets were resuspended in urea lysis buffer [8 M urea and 50 mM tris (pH 7.5) supplemented with 1:200 protease inhibitor cocktail (Merck)]. The cell wall was broken down by vortexing at high speed with ready-to-use glass beads (Scientific Industries) at 4°C for 10 min; 25 μl of 20% SDS was added followed by incubation at 95°C for 5 min. Samples were run on a 4 to 20% gradient gel (Bio-Rad) at a constant voltage of 100 V and then transferred to a nitrocellulose membrane using a semi-dry transfer machine (Bio-Rad). The membrane was blotted with α-HA (hemagglutinin) antibody at a dilution of 1:1000 (BioLegend) to detect either Rps2-HA-Avi or Rpl16a/b-HA-Avi followed by an IRDye 680LT goat α-mouse secondary antibody (LI-COR Biosciences) was used at 1:10,000 dilution. To detect the biotinylated ribosomal proteins, the membrane was blotted with 1:2000 Alexa Fluor 790 streptavidin (Jackson ImmunoResearch) in 3% bovine serum albumin. Membrane visualization was done by scanning with the Odyssey Imaging System (LI-COR Biosciences). For detecting sfGFP-Pex14 (fig. S2), a rabbit polyclonal primary antibody designed against GFP was used at a concentration of 1:1000 (Abcam), and a 790LT secondary goat α-rabbit (LI-COR Biosciences) was used at a dilution of 1:10,000. As a loading control, a mouse monoclonal primary antibody designed against actin was used at a concentration of 1:1000.

### Peroxisome proximity-specific ribosome profiling

Cells preparation is similar to that described in the previous section with the exception that, following the biotinylation assay, cells were harvested by filtration onto 0.45-μm pore size nitrocellulose filters (Whatman), scraped from the membrane, and immediately submerged in liquid nitrogen. The following steps of monosomes isolation, streptavidin pulldown of biotinylated ribosomes, and library generation were done as previously described (*21*, *33*, *34*).

#### Computational analysis

By comparing the ribosomal footprints obtained from the total ribosome fraction and the streptavidin-pulled fraction, ER-localized translation enrichment was measured.

#### Footprint sequence

Sequencing reads were demultiplexed and stripped of 3′ cloning adapters using in-house scripts. Reads were mapped sequentially to Bowtie indices composed of ribosomal RNAs, transfer RNAs, and finally all chromosomes using Bowtie 1.1.0. Only uniquely mapped, zero-mismatch reads from the final genomic alignment were used for subsequent analyses. These alignments were assigned a specific P-site nucleotide using a 15-nucleotide offset from the 3′ end of reads.

#### Gene enrichments

Gene-level enrichments were computed by taking the log_2_ ratio of biotinylated footprint density (reads per million) within a gene coding sequence (CDS) over the corresponding density of matched input ribosome profiling experiment. Yeast genes were excluded from all analysis if they met any of the following criteria: had fewer than 100 CDS-mapping footprints in the input sample of a particular experiment, annotated as “dubious” in the Saccharomyces Genome Database, and gene maps to the mitochondrial chromosome. In addition, regions where CDS overlaps another same-strand CDS were excluded from enrichment calculations. Genes that were enriched in the peroxisome-specific ribosomal profiling were found by comparing the enriched transcripts in Pex11-BirA and Pex25-BirA samples 2 or 5 min after biotin treatment to the total cell lysate under the same conditions. Log_2_ enrichments were separately normalized by subtracting the mean enrichment and dividing by the SD of enrichments for the corresponding experiment. Genes were then binned by the minimum number of sequencing counts. A gene where its log_2_ enrichment was above 0.5 was considered as enriched.

## Supplementary Materials

### This PDF file includes:

Figs. S1 to S13 Legend for table S1 Tables S2 and S3 Legend for movie S1 References

## References

[1] M. Veenhuis, J. M. Goodman, Peroxisomal assembly: Membrane proliferation precedes the induction of the abundant matrix proteins in the methyiotrophic yeast Candida boidinii. J. Cell. Sci. 6, 583–590 (1990). 10.1242/jcs.96.4.583 2283358

[2] V. D. Antonenkov, J. K. Hiltunen, Transfer of metabolites across the peroxisomal membrane. Biochim. Biophys. Acta 1822, 1374–1386 (2012). 22206997 10.1016/j.bbadis.2011.12.011

[3] R. Erdmann, W. Schliebs, Peroxisomal matrix protein import: The transient pore model. Nat. Rev. Mol. Cell Biol. 6, 738–742 (2005). 16103872 10.1038/nrm1710

[4] Y. Fujiki, S. Nagata, Peroxisome biogenesis and human peroxisome-deficiency disorders. Proc. Jpn. Acad. Ser. B Phys. Biol. Sci. 92, 463–477 (2016). 10.2183/pjab.92.463 PMC5328784 27941306

[5] M. Islinger, A. Voelkl, H. D. Fahimi, M. Schrader, The peroxisome: An update on mysteries 2.0. Histochem. Cell Biol. 150, 443–471 (2018). 30219925 10.1007/s00418-018-1722-5 PMC6182659

[6] F. D. Mast, R. A. Rachubinski, J. D. Aitchison, Peroxisome prognostications: Exploring the birth, life, and death of an organelle. J. Cell Biol. 219, e201912100 (2020). 32211898 10.1083/jcb.201912100 PMC7054992

[7] H. Rottensteiner, A. Kramer, S. Lorenzen, K. Stein, C. Landgraf, R. Volkmer-Engert, R. Erdmann, Peroxisomal membrane proteins contain common Pex19p-binding sites that are an integral part of their targeting signals. Mol. Biol. Cell 15, 3406–3417 (2004). 15133130 10.1091/mbc.E04-03-0188 PMC452593

[8] W. Girzalski, L. S. Hoffman, A. Schemenewitz, A. Nolte, W. H. Kunau, R. Erdmann, Pex19p-dependent targeting of Pex17p, a peripheral component of the peroxisomal protein import machinery. J. Biol. Chem. 28, 19417–19425 (2006). 10.1074/jbc.M603344200 16679311

[9] W. B. Snyder, K. N. Faber, T. J. Wenzel, A. Koller, G. H. Luers, L. Rangell, G. A. Keller, S. Subramani, Pex19p interacts with Pex3p and Pex10p and is essential for peroxisome biogenesis in *Pichia pastoris*. Mol. Biol. Cell 10, 1746–1761 (1999). 10.1091/mbc.10.6.1745 PMC25367 10359594

[10] A. Halbach, R. Rucktäschel, H. Rottensteiner, R. Erdmann, The N-domain of Pex22p can functionally replace the Pex3p N-domain in targeting and peroxisome formation. J. Biol. Chem. 6, 3906–3916 (2009). 10.1074/jbc.M806950200 19017643

[11] R. L. M. Jansen, I. J. van der Klei, The peroxisome biogenesis factors Pex3 and Pex19: Multitasking proteins with disputed functions. FEBS Lett. 593, 457–474 (2019). 30776093 10.1002/1873-3468.13340

[12] B. A. Cichocki, K. Krumpe, D. G. Vitali, D. Rapaport, Pex19 is involved in importing dually targeted tail-anchored proteins to both mitochondria and peroxisomes. Traffic 19, 770–785 (2018). 30033679 10.1111/tra.12604

[13] B. Schrul, W. Schliebs, Intracellular communication between lipid droplets and peroxisomes: The Janus face of PEX19. Biol. Chem. 399, 741–749 (2018). 29500918 10.1515/hsz-2018-0125

[14] L. Wrobel, U. Topf, P. Bragoszewski, S. Wiese, M. E. Sztolsztener, S. Oeljeklaus, A. Varabyova, M. Lirski, P. Chroscicki, S. Mroczek, E. Januszewicz, A. Dziembowski, M. Koblowska, B. Warscheid, A. Chacinska, Mistargeted mitochondrial proteins activate a proteostatic response in the cytosol. Nature 524, 485–488 (2015). 26245374 10.1038/nature14951

[15] E. A. Costa, K. Subramanian, J. Nunnari, J. S. Weissman, Defining the physiological role of SRP in protein-targeting efficiency and specificity. Science 359, 689–692 (2018). 29348368 10.1126/science.aar3607 PMC5970945

[16] O. Hermesh, R. P. Jansen, Take the (RN)A-train: Localization of mRNA to the endoplasmic reticulum. Biochim. Biophys. Acta 1833, 2519–2525 (2013). 23353632 10.1016/j.bbamcr.2013.01.013

[17] N. Aviram, M. Schuldiner, Targeting and translocation of proteins to the endoplasmic reticulum at a glance. J. Cell Sci. 130, 4079–4085 (2017). 29246967 10.1242/jcs.204396

[18] C. Lesnik, A. Golani-Armon, Y. Arava, Localized translation near the mitochondrial outer membrane: An update. RNA Biol. 12, 801–809 (2015). 26151724 10.1080/15476286.2015.1058686 PMC4615199

[19] V. A. Gold, P. Chroscicki, P. Bragoszewski, A. Chacinska, Visualization of cytosolic ribosomes on the surface of mitochondria by electron cryo-tomography. EMBO Rep. 18, 1786–1800 (2017). 28827470 10.15252/embr.201744261 PMC5623831

[20] R. Zoschke, R. Bock, Chloroplast translation: Structural and functional organization, operational control, and regulation. Plant Cell 30, 745–770 (2018). 29610211 10.1105/tpc.18.00016 PMC5969280

[21] C. C. Williams, C. H. Jan, J. S. Weissman, Targeting and plasticity of mitochondrial proteins revealed by proximity-specific ribosome profiling. Science 346, 748–751 (2014). 25378625 10.1126/science.1257522 PMC4263316

[22] G. Zipor, L. Haim-Vilmovsky, R. Gelin-Licht, N. Gadir, C. Brocard, J. E. Gerst, Localization of mRNAs coding for peroxisomal proteins in the yeast, Saccharomyces cerevisiae. Proc. Natl. Acad. Sci. U.S.A. 106, 19848–19853 (2009). 19903887 10.1073/pnas.0910754106 PMC2785255

[23] P. Lill, T. Hansen, D. Wendscheck, B. U. Klink, T. Jeziorek, D. Vismpas, J. Miehling, J. Bender, A. Schummer, F. Drepper, W. Girzalsky, B. Warscheid, R. Erdmann, C. Gatsogiannis, Towards the molecular architecture of the peroxisomal receptor docking complex. Proc. Natl. Acad. Sci. U.S.A. 117, 33216–33224 (2021). 10.1073/pnas.2009502117 PMC7776778 33323485

[24] M. Komori, S. W. Rasmussen, J. A. K. W. Kiel, R. J. S. Baerends, J. M. Cregg, I. J. Van Der Klei, M. Veenhuis, The *Hansenula polymorpha PEX14* gene encodes a novel peroxisomal membrane protein essential for peroxisome biogenesis. EMBO J. 16, 44–53 (1997). 9009266 10.1093/emboj/16.1.44 PMC1169612

[25] E. Yifrach, S. Fischer, S. Oeljeklaus, M. Schuldiner, E. Zalckvar, B. Warscheid, Defining the mammalian peroxisomal proteome, in *Subcellular Biochemistry* (Springer, 2018), pp. 47–66. 10.1007/978-981-13-2233-4_2 30378018

[26] K. Knoops, S. Manivannan, M. N. Cepińska, A. M. Krikken, A. M. Kram, M. Veenhuis, I. J. van der Klei, Preperoxisomal vesicles can form in the absence of Pex3. J. Cell Biol. 204, 659–668 (2014). 24590171 10.1083/jcb.201310148 PMC3941047

[27] A. Maekiniemi, R. H. Singer, E. Tutucci, Single molecule mRNA fluorescent in situ hybridization combined with immunofluorescence in *S. cerevisiae*: Dataset and quantification. Data Brief 30, 105511 (2020). 32368581 10.1016/j.dib.2020.105511 PMC7186551

[28] F. Gallardo, P. Chartrand, Visualizing mRNAs in fixed and living yeast cells. Methods Mol. Biol. 714, 203–219 (2011). 21431743 10.1007/978-1-61779-005-8_13

[29] H. Wu, R. de Boer, A. M. Krikken, A. Akşit, W. Yuan, I. J. van der Klei, Peroxisome development in yeast is associated with the formation of Pex3-dependent peroxisome-vacuole contact sites. Biochim. Biophys. Acta Mol. Cell. Res. 1866, 349–359 (2019). 30595161 10.1016/j.bbamcr.2018.08.021

[30] K. Knoops, R. De Boer, A. Kram, I. J. Van Der Klei, Yeast pex1 cells contain peroxisomal ghosts that import matrix proteins upon reintroduction of Pex1. J. Cell Biol. 211, 955–962 (2015). 26644511 10.1083/jcb.201506059 PMC4674281

[31] W. Kukulski, M. Schorb, M. Kaksonen, J. A. G. Briggs, Plasma membrane reshaping during endocytosis is revealed by time-resolved electron tomography. Cell 150, 508–520 (2012). 22863005 10.1016/j.cell.2012.05.046

[32] C. H. Jan, C. C. Williams, J. S. Weissman, Principles of ER cotranslational translocation revealed by proximity-specific ribosome profiling. Science 346, 1257521 (2014). 25378630 10.1126/science.1257521 PMC4285348

[33] N. Aviram, T. Ast, E. A. Costa, E. C. Arakel, S. G. Chuartzman, C. H. Jan, S. Haßdenteufel, J. Dudek, M. Jung, S. Schorr, R. Zimmermann, B. Schwappach, J. S. Weissman, M. Schuldiner, The SND proteins constitute an alternative targeting route to the endoplasmic reticulum. Nature 540, 134–138 (2016). 27905431 10.1038/nature20169 PMC5513701

[34] R. S. Hamilton, I. Davis, Identifying and searching for conserved RNA localisation signals. Methods Mol. Biol. 714, 447–466 (2011). 21431757 10.1007/978-1-61779-005-8_27 PMC3082378

[35] H. Rottensteiner, K. Stein, E. Sonnenhol, R. Erdmann, Conserved function of Pex11p and the novel Pex25p and Pex27p in peroxisome biogenesis. Mol. Biol. Cell 14, 4316–4328 (2003). 14517338 10.1091/mbc.E03-03-0153 PMC207022

[36] D. Zabezhinsky, B. Slobodin, D. Rapaport, J. E. Gerst, An essential role for COPI in mRNA localization to mitochondria and mitochondrial function. Cell Rep. 15, 540–549 (2016). 27068463 10.1016/j.celrep.2016.03.053

[37] S. Gabay-Maskit, L. Daniel Cruz-Zaragoza, N. Shai, M. Eisenstein, C. Bibi, N. Cohen, T. Hansen, E. Yifrach, N. Harpaz, R. Belostotsky, W. Schliebs, M. Schuldiner, R. Erdmann, E. Zalckvar, A piggybacking mechanism enables peroxisomal localization of the glyoxylate cycle enzyme Mdh2 in yeast. J. Cell Sci. 133, jcs244376 (2020). 33177075 10.1242/jcs.244376 PMC7758625

[38] A. M. Valm, S. Cohen, W. R. Legant, J. Melunis, U. Hershberg, E. Wait, A. R. Cohen, M. W. Davidson, E. Betzig, J. Lippincott-Schwartz, Applying systems-level spectral imaging and analysis to reveal the organelle interactome. Nature 546, 162–167 (2017). 28538724 10.1038/nature22369 PMC5536967

[39] N. Shani, D. Valle, A Saccharomyces cerevisiae homolog of the human adrenoleukodystrophy transporter is a heterodimer of two half ATP-binding cassette transporters. Proc. Natl. Acad. Sci. U.S.A. 93, 11901–11906 (1996). 8876235 10.1073/pnas.93.21.11901 PMC38156

[40] N. Shani, P. A. Watkins, D. Vallett, J. W. Littlefield, J. Hopkins, PXA1, a possible Saccharomyces cerevisiae ortholog of the human adrenoleukodystrophy gene. Proc. Natl. Acad. Sci. U.S.A. 92, 6012–6016 (1995). 7597071 10.1073/pnas.92.13.6012 PMC41632

[41] M. Hanscho, D. E. Ruckerbauer, N. Chauhan, H. F. Hofbauer, S. Krahulec, B. Nidetzky, S. D. Kohlwein, J. Zanghellini, K. Natter, Nutritional requirements of the BY series of Saccharomyces cerevisiae strains for optimum growth. FEMS Yeast Res. 12, 796–808 (2012). 22780918 10.1111/j.1567-1364.2012.00830.x

[42] R. D. Gietz, R. A. Woods, Transformation of yeast by lithium acetate/single-stranded carrier DNA/polyethylene glycol method. Methods Enzymol. 350, 87–96 (2002). 12073338 10.1016/s0076-6879(02)50957-5

[43] I. Yofe, M. Schuldiner, Primers-4-Yeast: A comprehensive web tool for planning primers for *Saccharomyces cerevisiae*. Yeast 31, 77–80 (2014). 24408512 10.1002/yea.2998

[44] A. Raj, P. van den Bogaard, S. A. Rifkin, A. van Oudenaarden, S. Tyagi, Imaging individual mRNA molecules using multiple singly labeled probes. Nat. Methods 5, 877–879 (2008). 18806792 10.1038/nmeth.1253 PMC3126653

[45] T. Lionnet, K. Czaplinski, X. Darzacq, Y. Shav-Tal, A. L. Wells, J. A. Chao, H. Y. Park, V. de Turris, M. Lopez-Jones, R. H. Singer, A transgenic mouse for in vivo detection of endogenous labeled mRNA. Nat. Methods 8, 165–170 (2011). 21240280 10.1038/nmeth.1551 PMC3076588

[46] J. Schindelin, I. Arganda-Carreras, E. Frise, V. Kaynig, M. Longair, T. Pietzsch, S. Preibisch, C. Rueden, S. Saalfeld, B. Schmid, J. Y. Tinevez, D. J. White, V. Hartenstein, K. Eliceiri, P. Tomancak, A. Cardona, Fiji: An open-source platform for biological-image analysis. Nat. Methods 9, 676–682 (2012). 22743772 10.1038/nmeth.2019 PMC3855844

[47] Y. S. Bykov, N. Cohen, N. Gabrielli, H. Manenschijn, S. Welsch, P. Chlanda, W. Kukulski, K. R. Patil, M. Schuldiner, J. A. G. Briggs, High-throughput ultrastructure screening using electron microscopy and fluorescent barcoding. J. Cell Biol. 218, 2797–2811 (2019). 31289126 10.1083/jcb.201812081 PMC6683748

[48] J. R. Kremer, D. N. Mastronarde, J. R. McIntosh, Computer visualization of three-dimensional image data using IMOD. J. Struct. Biol. 116, 71–76 (1996). 8742726 10.1006/jsbi.1996.0013

[49] S. Berg, D. Kutra, T. Kroeger, C. N. Straehle, B. X. Kausler, C. Haubold, M. Schiegg, J. Ales, T. Beier, M. Rudy, K. Eren, J. I. Cervantes, B. Xu, F. Beuttenmueller, A. Wolny, C. Zhang, U. Koethe, F. A. Hamprecht, A. Kreshuk, ilastik: Interactive machine learning for (bio)image analysis. Nat. Methods 16, 1226–1232 (2019). 31570887 10.1038/s41592-019-0582-9

[50] U. Weill, I. Yofe, E. Sass, B. Stynen, D. Davidi, J. Natarajan, R. Ben-Menachem, Z. Avihou, O. Goldman, N. Harpaz, S. Chuartzman, K. Kniazev, B. Knoblach, J. Laborenz, F. Boos, J. Kowarzyk, S. Ben-Dor, E. Zalckvar, J. M. Herrmann, R. A. Rachubinski, O. Pines, D. Rapaport, S. W. Michnick, E. D. Levy, M. Schuldiner, Genome-wide SWAp-Tag yeast libraries for proteome exploration. Nat. Methods 15, 617–622 (2018). 29988094 10.1038/s41592-018-0044-9 PMC6076999

[51] F. Madeira, Y. M. Park, J. Lee, N. Buso, T. Gur, N. Madhusoodanan, P. Basutkar, A. R. N. Tivey, S. C. Potter, R. D. Finn, R. Lopez, The EMBL-EBI search and sequence analysis tools APIs in 2019. Nucleic Acids Res. 47, W636–W641 (2019). 30976793 10.1093/nar/gkz268 PMC6602479

[52] U. Weill, N. Cohen, A. Fadel, S. Ben-Dor, M. Schuldiner, Protein topology prediction algorithms systematically investigated in the yeast *Saccharomyces cerevisiae*. Bioessays 41, 1800252 (2019). 10.1002/bies.201800252 PMC7611422 31297843

