# Supplementary material for: Peroxisome function relies on organelle-associated mRNA translation
Source: Sci Adv. 2022 Jan 12;8(2):eabk2141. doi: 10.1126/sciadv.abk2141 (PMC8754406; doi:10.1126/sciadv.abk2141)
Supplement: Supplementary file 1 — Figs. S1 to S13 Legend for table S1 Tables S2 and S3 Legend for movie S1 References [file sciadv.abk2141_sm.pdf]

Science Advances
AAAS

Supplementary Materials for

Peroxisome function relies on organelle-associated mRNA translation

Noa Dahan*, Yury S. Bykov, Elizabeth A. Boydston, Amir Fadel,
Zohar Gazi, Hodaya Hochberg-Laufer, James Martenson, Vlad Denic, Yaron Shav-Tal,
Jonathan S. Weissman, Naama Aviram*, Einat Zalckvar, Maya Schuldiner*

*Corresponding author. Email: maya.schuldiner@weizmann.ac.il (M.S.); noadahan.mail@gmail.com (N.D.); naviram@rockefeller.edu (N.A.)

Published 12 January 2022, *Sci. Adv.* **8**, eabk2141 (2022)
DOI: 10.1126/sciadv.abk2141

The PDF file includes:

Figs. S1 to S13
Legend for table S1
Tables S2 and S3
Legend for movie S1
References

Other Supplementary Material for this manuscript includes the following:

Table S1
Movie S1

Supplementary figures

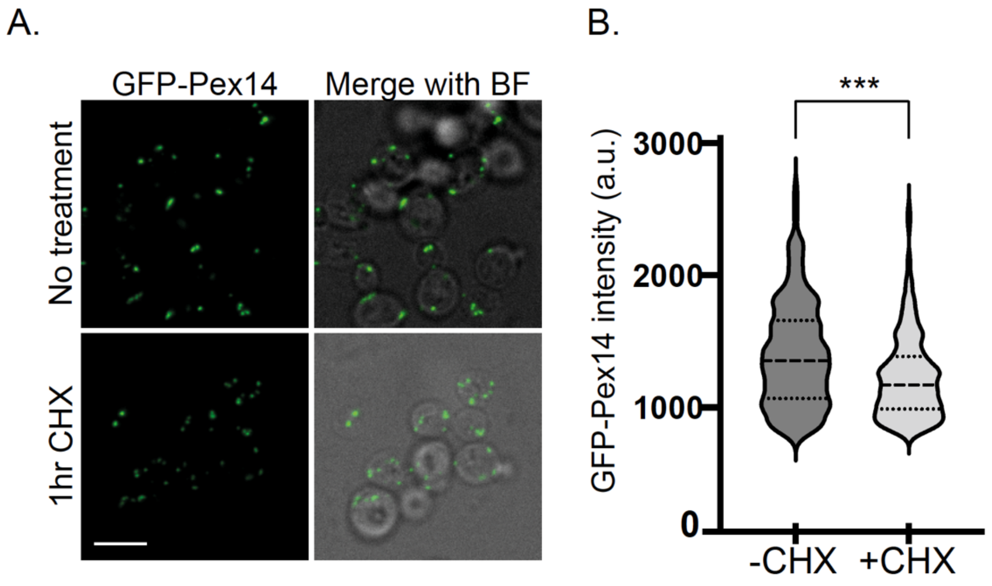

Fig. S1. Treatment of cells with Cycloheximide (CHX) for 60 minutes reduces the synthesis of GFP-Pex14.

(A) Microscopic images of yeast cells expressing GFP-Pex14 (green) before and after 1 hour of CHX treatment. The intensity of the GFP is reduced after 1 hour of CHX treatment. Bar, 5 µm.

(B) The intensity of GFP-Pex14 signal was measured for 500 peroxisomes and plotted on a violin graph (***, Unpaired t-test, $P<0.05$)

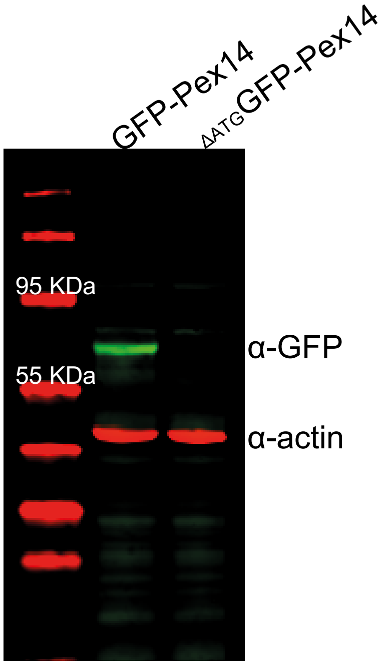

Fig. S2. Removal of the starter methionine (ATG) of GFP abolishes GFP-Pex14 expression. A western blot analysis of cells expressing either GFP-Pex14 with or without (ΔATG) the starter methionine under the *NOP1* promoter integrated into the HO locus. The blots were incubated with α-GFP antibody showing the expected size of about 75kDa of the fused GFP to Pex14 (green). An α-actin antibody was used as the loading control (red).

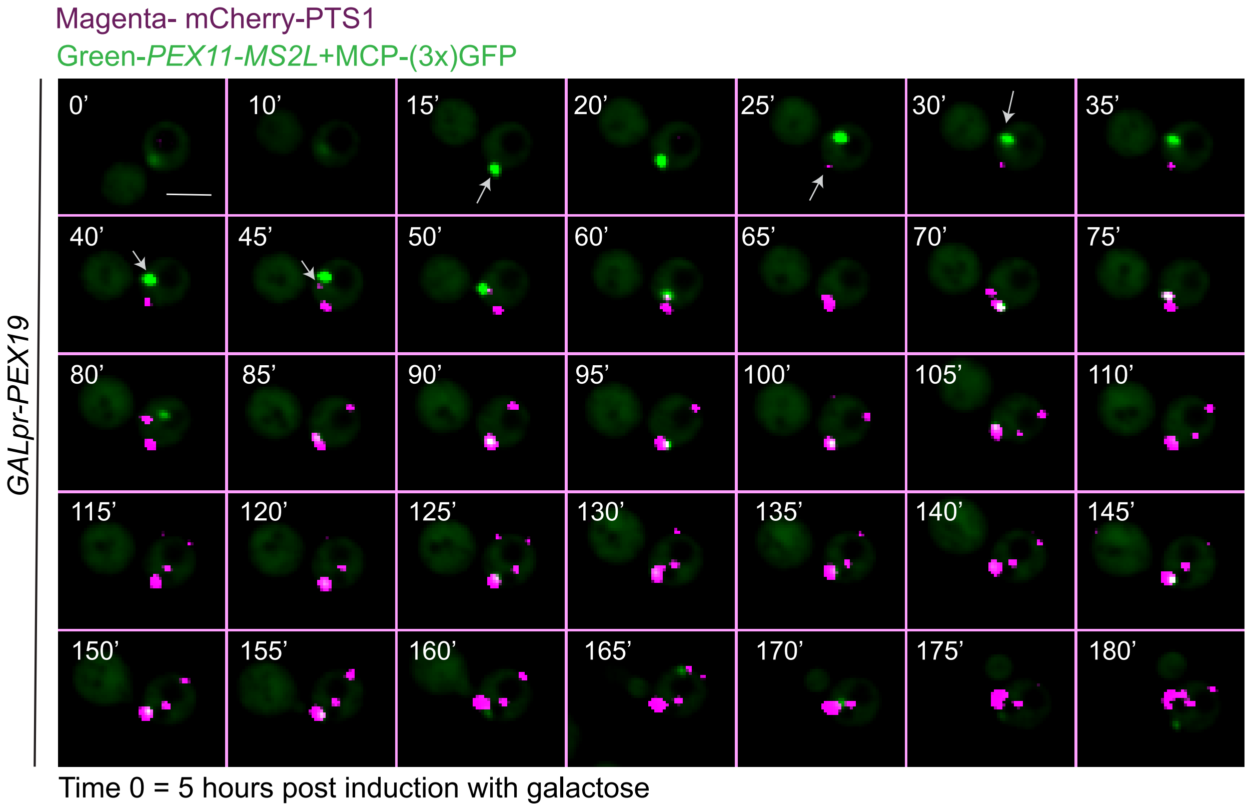

Figure. S3 Peroxisomal mRNA granule localization precedes the formation of a mature peroxisome.

In vivo time lapse of mRNA granules of *PEX11*-MS2L detected by MCP-3(x)GFP upon Pex19 induction with the *GALpr* induction system. Mature Peroxisomes are marked by mCherry-PTS1 and begin forming 5 hours post GAL induction. Images were taken every 5 minutes. Arrows indicate the area where a peroxisome was detected following *PEX11*-mRNA presence. The mRNA signal is very strong before peroxisome formation and during the maturation process and is decreased in intensity following division of the nascent peroxisome. Bar, 5 µm.

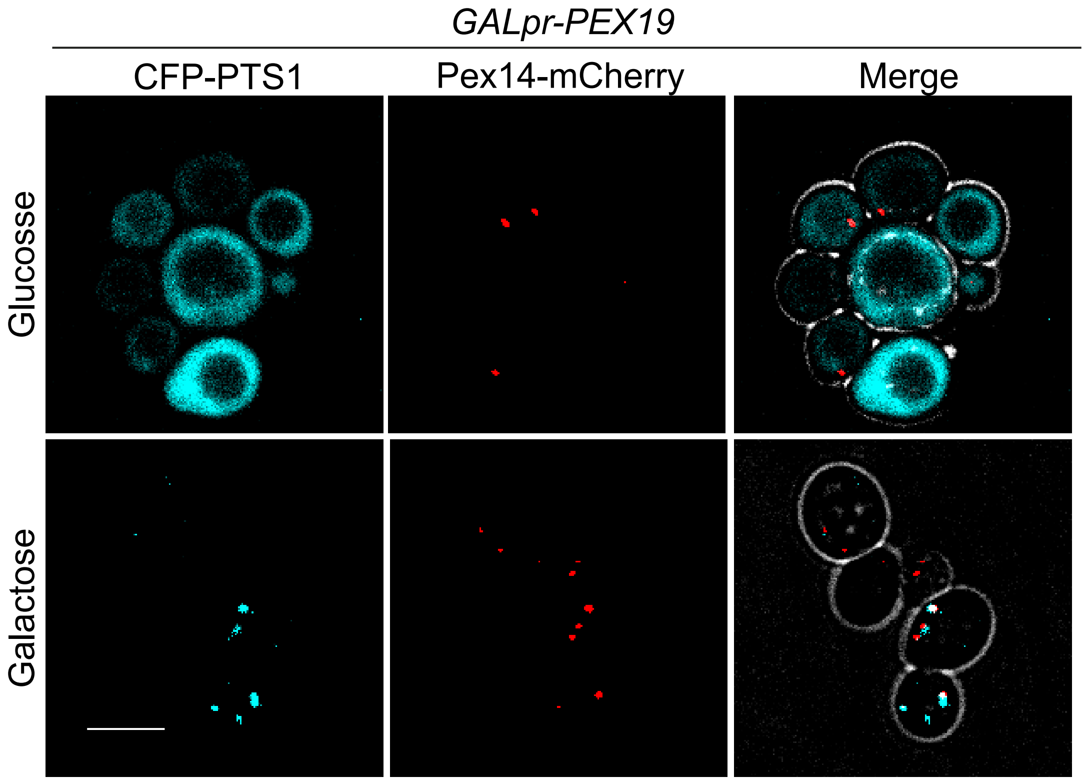

Fig S4. Immature peroxisomes are visible in the absence of Pex19.

A representative micrograph showing the expression of Pex14-mCherry, often used as a pre peroxisomal vesicle marker, in a punctate pattern prior to *GALpr-PEX19* induction. Mature peroxisomes are marked by CFP fused to a peroxisomal targeting signal 1, PTS1 (cyan) and are correctly targeted to peroxisomes only in the presence of Pex19, when cells are grown on Galactose. Bar, 5 µm

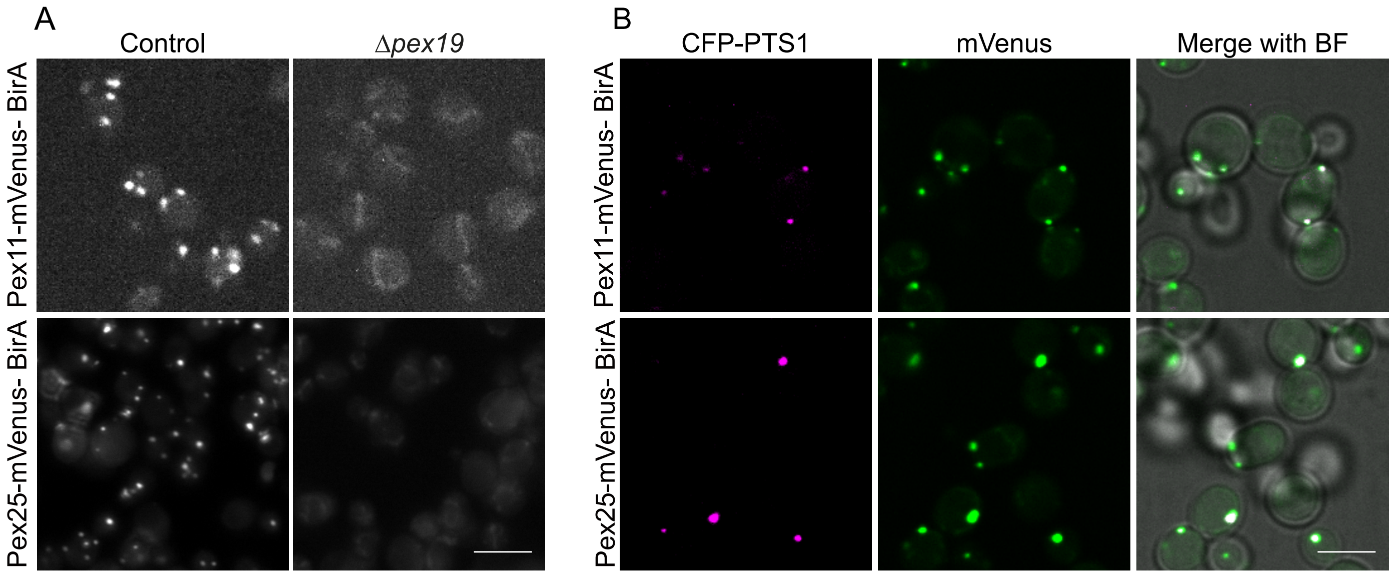

Fig. S5. Pex25-mVenus-BirA and Pex11-mVeus-BirA are correctly localized to peroxisomes.

(A) Micrographs showing the punctate pattern of Pex11-mVenus-BirA and Pex25-mVenus-BirA (green) that disappear upon *pex19* deletion. **(B)** Micrographs showing colocalization between a mature peroxisome marker CFP-PTS1 (magenta) and Pex11-mVenus-BirA or Pex25-mVenus-BirA (green). Both panels demonstrate correct localization of the peroxisomal membrane proteins that were used for the proximity labeling assays performed in figure 3 in the main text. Bar, 5 μm

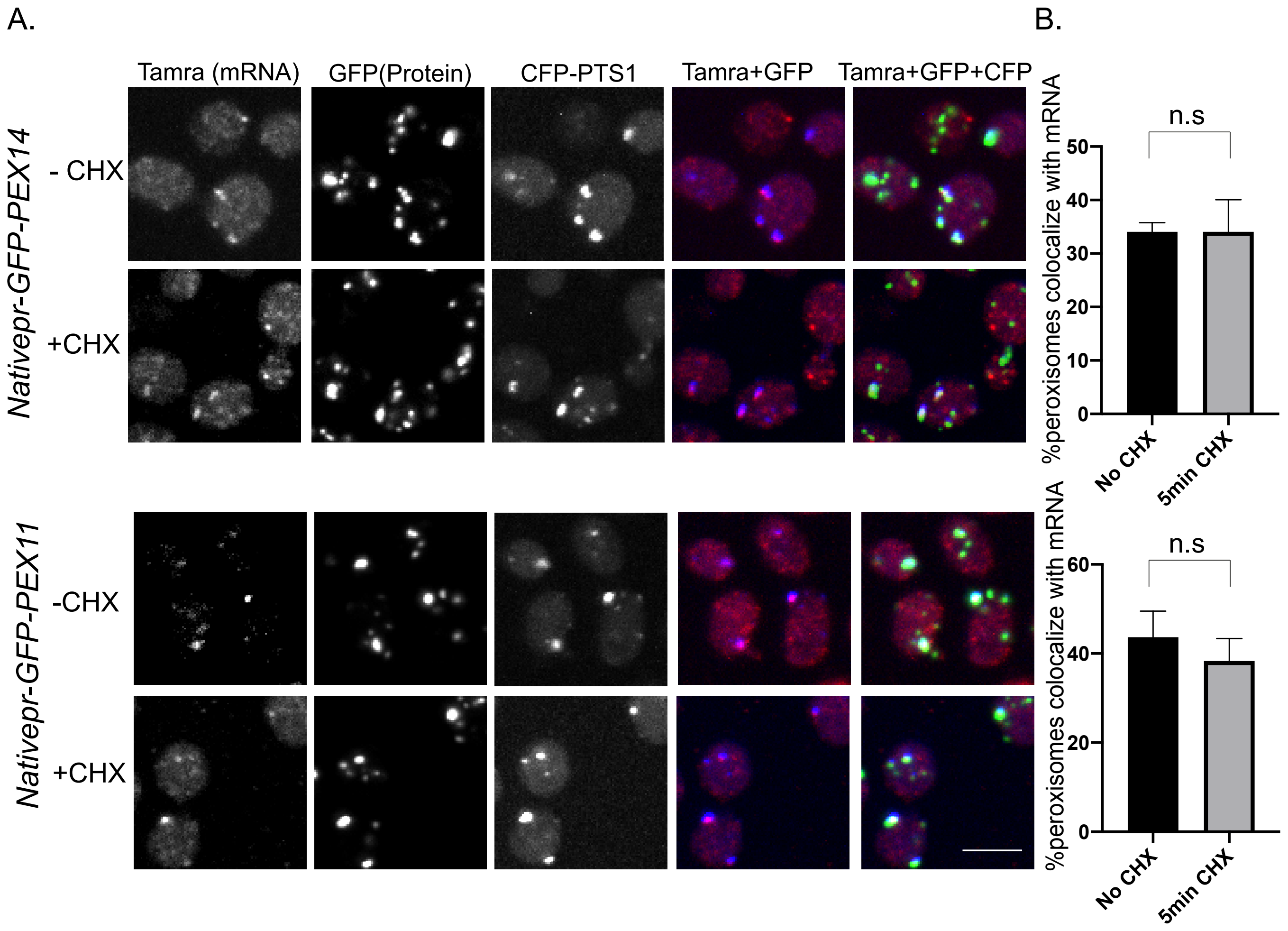

Fig. S6. The enrichment of locally translated peroxisomal membrane proteins is not affected by the addition of the translational inhibitor, Cycloheximide (CHX).

(A) Representative micrographs of maximal intensity projections following smRNA-FISH of *GFP-PEX14* and *GFP-PEX11* expressing yeast strains. Peroxisomes are marked by CFP-PTS1. Samples were subject to smFISH with and without the addition of CHX, 5 minutes prior to fixation. Bar, 5 µm. **(B)** The percentage of peroxisomes that co-localize with the smRNA-FISH fluorescent signal were counted and are presented by the bar graphs to the right of the corresponding micrographs (*n=50*, P>0.05, unpaired *t*-test).

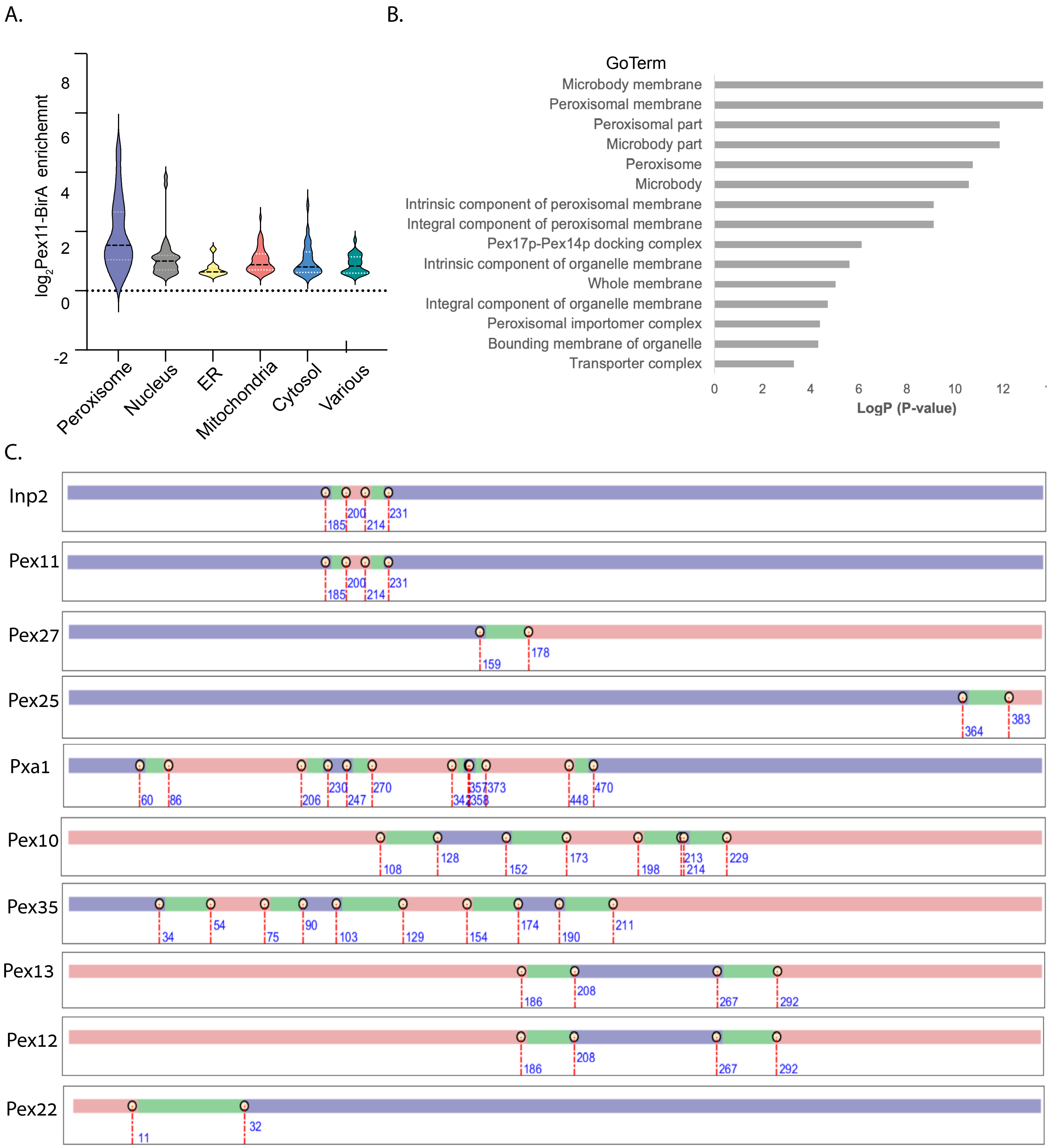

Fig. S7. The peroxisomal specific ribosome profiling is enriched for transcripts that encodes for peroxisomal membrane proteins. (A) peroxisomal transcripts were enriched by PRP. 40 transcripts that were enriched by Pex11-BirA after 2 minutes biotin pulse were plotted on a violin plot according to their cellular localization, median enrichment levels are marked by black dashed

line, which is highest for peroxisomal transcripts. **(B) GO term enrichment analysis of locally synthesized peroxisomal proteins**. A bar graph demonstrating the enrichment of transcripts translated in proximity to peroxisomes using their Gene Ontology (GO) term of "localization". The graph represents analysis of the 40 transcripts that were enriched by four independent experiments (with either Pex25-BirA or Pex11-BirA following either 2 or 5 minutes CHX pulse) analyzed compared to all expressed genes in *S. cerevisiae*. (**C**) **77% of enriched transcripts are predicted to be peroxisomal membrane proteins**. Presented are protein illustrations of 10 out of 13 highly PRP enriched transcripts. Green regions are predicted to form a transmembrane domain (TMD). In pink are cytosolic facing regions and in purple are regions facing the lumen of peroxisomes. Each region is defined by the position of encompassing amino acids as marked by blue numbers. TMDs were Predicted by the PolyPhobius algorithm and visualized by the TopologYeast web tool (*52*).

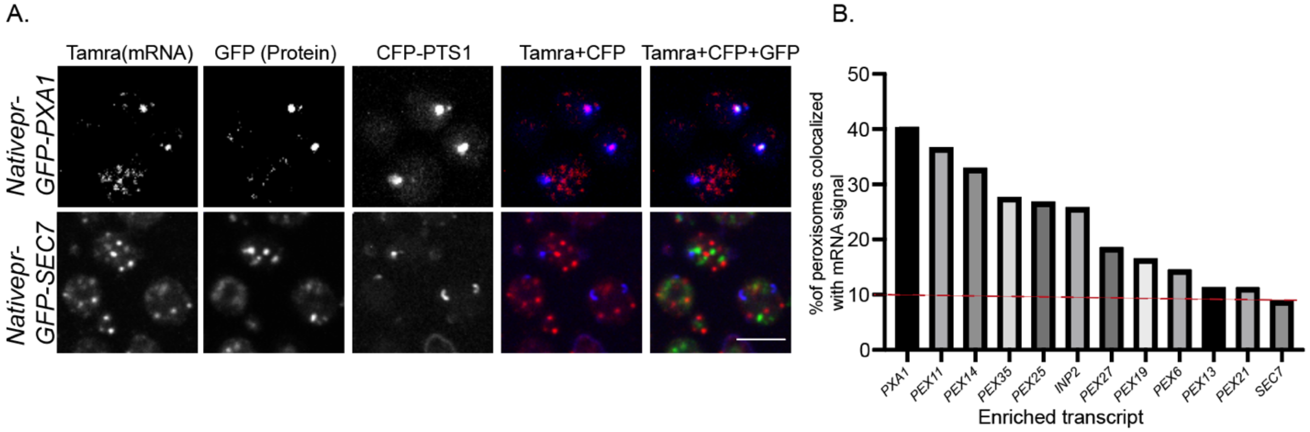

Fig S8. Transcripts enriched in the peroxisome-specific ribosome profiling colocalize with peroxisomes.

(A) An example of maximum intensity projections of fluorescent microscopy images following single molecule (sm) RNA-FISH of *GFP-PXA1* expressed under its native promoter. *GFP-PXA1* mRNA is shown in red (following hybridization with Stellaris®RNA FISH probes conjugated to TAMRA fluorescent dye), GFP-Pxa1 protein is shown in green, Peroxisomes are labeled by a Cyan Fluorescent Protein (CFP) fused to a Peroxisomal Targeting Signal (PTS1) shown in blue. Bar, 5 µm. **(B)** A bar graph summarizing the analysis of a comprehensive smRNA-FISH experiment in which the percentage of peroxisomes that colocalized with mRNA molecules of all transcripts that were highly enriched by the peroxisome specific ribosome profiling assay was counted. The Golgi protein Sec7 was used as a negative control. Bar, 5 µm, *n=100*.

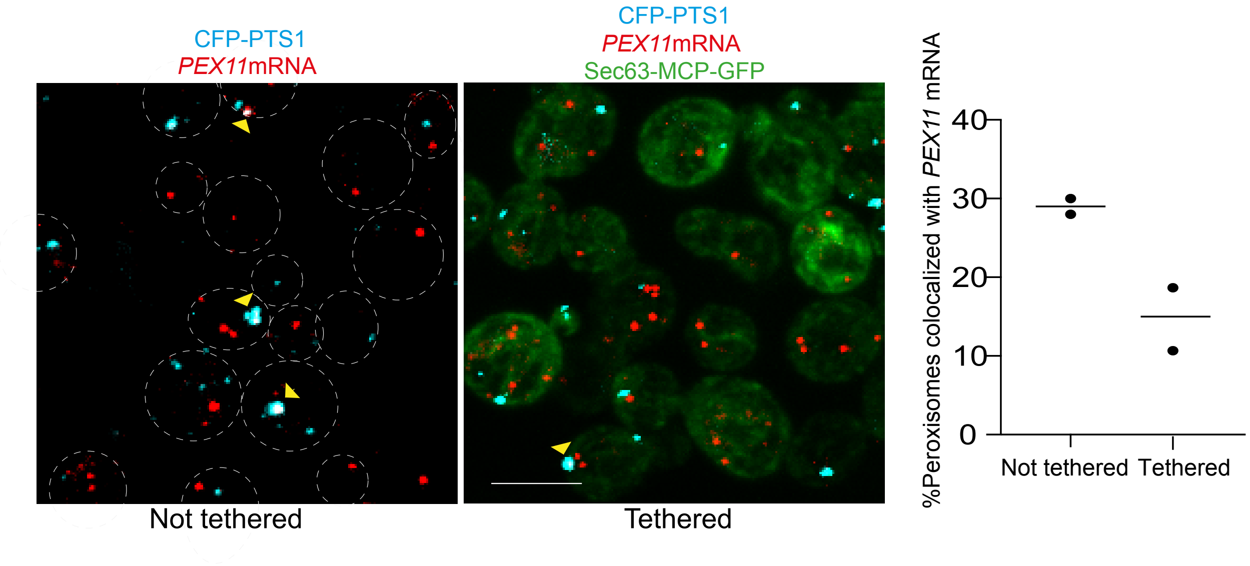

Fig. S9. ER tethering of *PEX11*-MS2L results in lower percentage of peroxisomes colocalizing with *PEX11*-MS2L mRNA.

A representative micrograph of smRNA-FISH experiment of strains with *PEX11*-MS2L and a marker for mature peroxisomes (cyan). Addition of an ER tethering element (Sec63-MCP, green) reduced the percentage of peroxisomes that colocalized with *PEX11* mRNA. Areas of colocalization are marked by yellow arrowheads and quantified in the graph. *n=150* (150 cells were counted for their signals), each dot stands for a biological repeat. Bar, 5 µm.

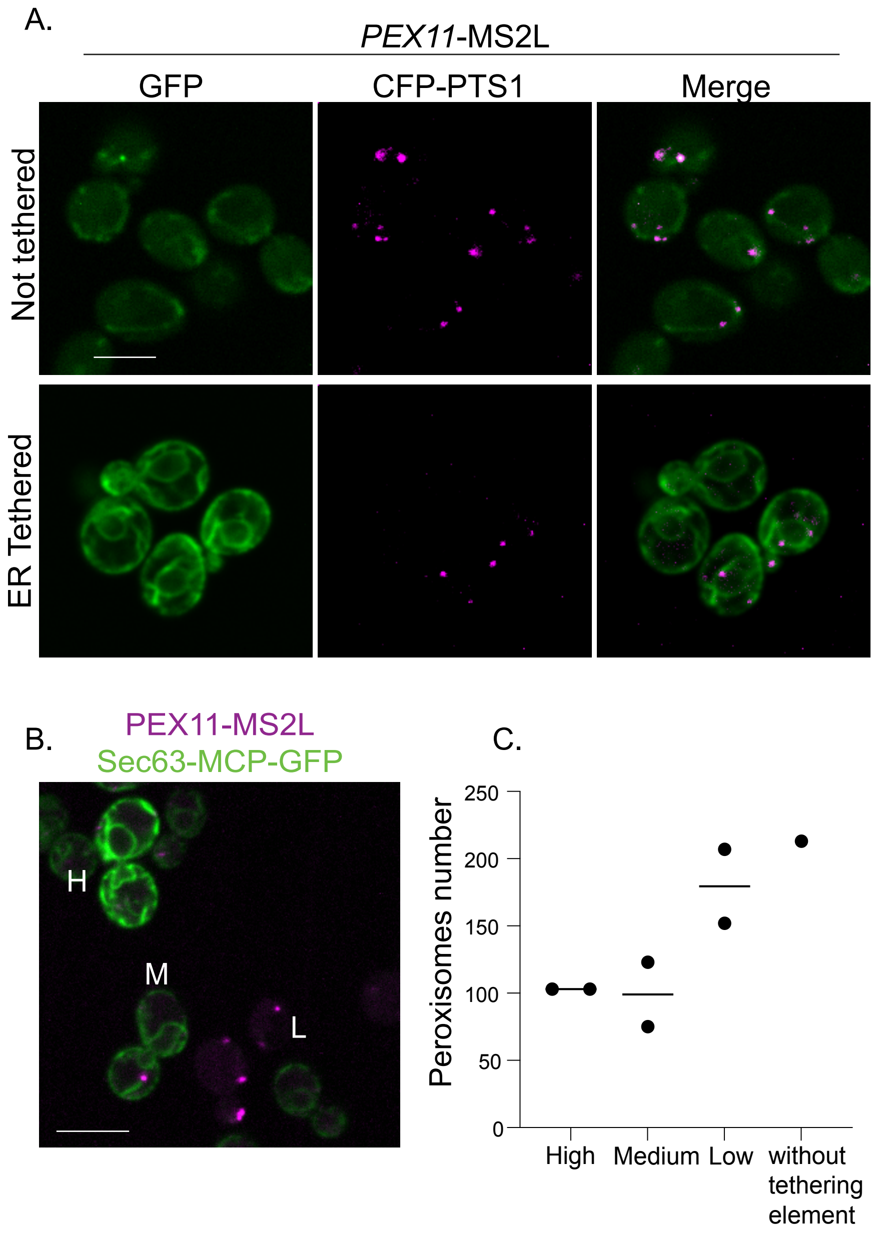

Fig. S10. ER tethering of *PEX11*-MS2L caused a reduction in mature peroxisome number. **A.** A representative micrograph of strains with *PEX11*-MS2L. Upon addition of the tethering element a reduction in mature peroxisomes, marked by CFP-PTS1 (magenta) number was detected. (**B**) The number of peroxisomes decreases the stronger the tethering of *PEX11* mRNA to the ER. Peroxisomes are marked by CFP-PTS1 (magenta) in cells expressing *PEX11*-MS2L that is mis-localized to the ER by the episomally expressed tethering element made of Sec63-MCPx2-GFP (green). Cells were visually divided into three subgroups that differ in the expression levels of the tethering element: High (H), Medium (M) and Low (L). (**C**) The number of peroxisomes was quantified for each group. Bar, 5 µm, *n=100*.

PEX14-MS2L
Om45-2xMCP-GFP

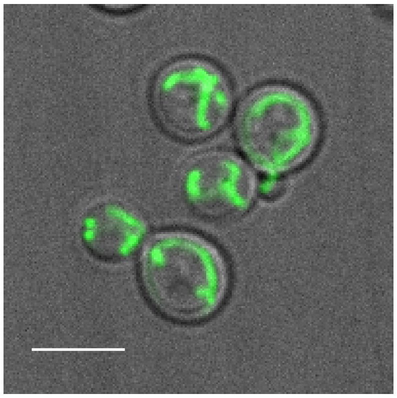

Fig. S11. Om45 is correctly targeted to mitochondria when fused to MCP(x2)-GFP. Fluorescent micrograph showing that OM45-MCP-GFP (expressed in cells in which *PEX14* mRNA is tagged with MS2L) is correctly localized to mitochondria. Bar, 5 µm

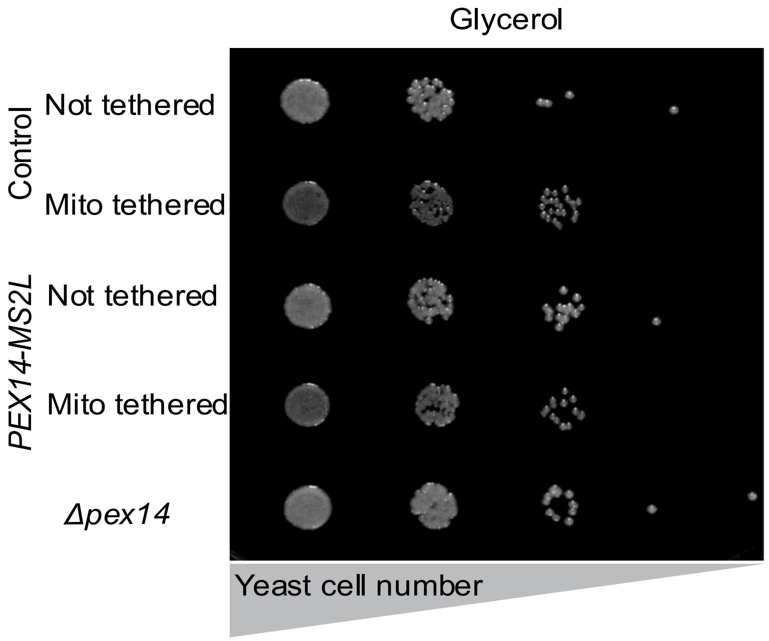

Fig. S12. Mislocalization of *PEX14* mRNA does not have a toxic effect on respiration. Drop dilution assay of cells grown for 5 days on agar plates with glycerol as a sole carbon source. The mitochondrial tethered strain (*PEX14MS2L*/ OM45-MCPx2-GFP) shows normal growth, similar to control.

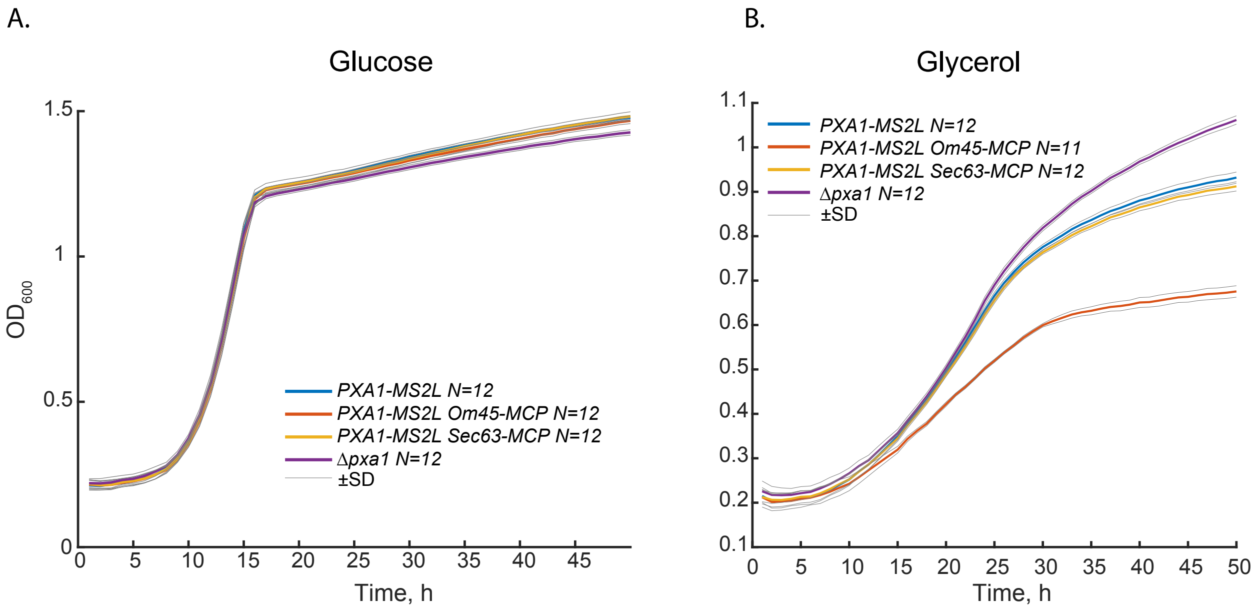

Fig S13. Mis localization of *PXA1-MS2L* transcript to the mitochondria has a toxic effect on respiration. Growth curves obtained using a plate reader, comparing the growth kinetics of yeast strains in which *PXA1* transcripts were either not tethered (PXA1-MS2L), tethered to mitochondria (*PXA1-MS2L* Om45-CP), tethered to the ER (*PXA1*-MS2L, Sec63-MCP) or deleted (*pxa1*). Cells were grown in media containing either fermentable glucose (**A**) or non-fermentable glycerol (**B**) as a sole carbon source. *N*, number of wells.

Table S1. A list of enriched transcripts by peroxisome specific ribosome profiling. Comprehensive list of transcripts that were enriched in the peroxisome specific ribosome profiling assay in all four assays, when Pex11-BirA or Pex25-BirA is a biotin donor after either 2 or 5 minutes CHX treatment. Table S1 is available in the online version of the manuscript.

Table S2. List of yeast strains used in this study.				
**Strain Number**	**Strain Name**	**Genotype**	**Comments**	**Mating Type**
YMS701	BY4741	*his3Δ1 leu2Δ0 met15Δ0 ura3Δ0*		a
YMS6010	sfGFP-Pex14+CFP-PTS1	*his3Δ1 leu2Δ0 met15Δ0 ura3Δ0 lys+ can1Δ::GAL1pr-SceI::STE2pr-SpHIS5 lyp1Δ::STE3pr-LEU2 NATIVEpr-sfGFP-PEX14*		α
YMS6022	sfGFP-Sec7+CFP-PTS1	*his3Δ1 leu2Δ0 met15Δ0 ura3Δ0 lys+ can1Δ::GAL1pr-SceI::STE2pr-SpHIS5 lyp1Δ::STE3pr-LEU2 NATIVEpr-sfGFP-SEC7*		α
YMS5093	sfGFP-Pex14	*his3Δ1 leu2Δ0 met15Δ0 ura3Δ0 URA3∷NOP1pr-SfGFP-PEX14,*		a
YMS5094	*noATG*_sfGFP-Pex14	*his3Δ1 leu2Δ0 met15Δ0 ura3Δ0; URA3∷NOP1pr-ΔatgSfGFP-PEX14*		a
YMS4472	*PEX11-MS2L* x MCP-GFP(x3)	*his3Δ1 leu2Δ0 met15Δ0 ura3Δ0 PEX11:loxP::MS2L::PEX11_3'UTR,*	Described in (*22*) Transformed with plasmid pMS196	a
YMS4473	*PEX14-MS2L* x MCP-GFP(x3)	*his3Δ1 leu2Δ0 met15Δ0 ura3Δ0 PEX14:loxP::MS2L::PEX14_3'UTR,*	Described in (*22*). Transformed with plasmid pMS196	a
YMS4678	*PEX11-MS2L x* MCP-GFP(x3) x *GALpr-PEX19* x mCherry-PTS1	*his3Δ1 leu2Δ0 met15Δ0 ura3Δ0 PEX11::loxP::MS2L::PEX11_3'UTR, G418∷Galpr-PEX19*	The strain was transformed with pMS196 and pMS617	a
YMS5939	*PEX11-MS2L* x MCP-GFP(x3) x *GALpr-PEX19* x Pex14-mCherry x CFP-PTS1	*his3Δ1 leu2Δ0 met15Δ0 ura3Δ0, PEX11::loxP::MS2L::PEX11_3'UTR, G418-Galpr-PEX19, PEX14-mCherry-NAT*	The strain was transformed with pMS196 and pMS618	a
YMS6021	mCherry-Pex14	*his3Δ1 leu2Δ0 met15Δ0 ura3Δ0 can1Δ::GAL1pr-SceI::STE2pr-SpHIS5 lyp1Δ::STE3pr-LEU2 NAT::TEF2pr-mCherry-PEX14*	Used for CLEM	α
YMS2331	Rpl16a/b-HA-TEV-AVI	*RPL16a/b- HA-TEV-AVI ura3Δ0 lys2Δ0 his3Δ1 leu2Δ0*	Described in (21,32)	a

YMS3821	Sec63-mVenus-BirA x Rpl16a/b-HA-TEV-AVI	*ura3Δ0 lys2Δ0 his3Δ1 leu2Δ0 RPL16a/b- HA-TEV-AVI SEC63-mVenus-BirA-HIS5*	Described in: *(32)*	a
YMS2842	Pex11-mVenus-BirA x Rpl16a/b-HA-TEVAVI	*ura3Δ0 lys2Δ0 his3Δ1 leu2Δ0 RPL16a/b- HA-TEV-AVI PEX11-mVenus-BirA-HIS5*		a
YMS2844	Pex25-mVenus-BirA x Rpl16a/b-HA-TEVAVI	*ura3Δ0 lys2Δ0 his3Δ1 leu2Δ0 RPL16a/b- HA-TEV-AVI PEX25-mVenus-BirA-HIS5*		a
YMS2794	RPS2-HA-TEVAVI	*leu2Δ0 ura3Δ0 met15Δ0 his3Δ1 RPS2-HA-TEV-AVI*	Described in:(*32)*	a
YMS2845	Pex25-mVenus-BirA x RPS2-HA-TEVAVI	*leu2Δ0 ura3Δ0 met15Δ0 his3Δ1 RPS2-HA-TEV-AVI PEX25-mVenus-BirA-HIS5*		a
YMS2843	Pex11-mVenus-BirA x RPS2-HA-TEVAVI	*leu2Δ0 ura3Δ0 met15Δ0 his3Δ1 RPS2-HA-TEV-AVI PEX11-mVenus-BirA-HIS5*		a
YMS4178	Tef2-mVenus-BirA x RPS2-HA-TEVAVI	*leu2Δ0 ura3Δ0 met15Δ0 his3Δ1 RPS2-HA-TEV-AVI TEF2-mVenus-BirA-HIS5*		a
YMS6011	sfGFP-Pex11 *x* CFP-PTS1	*his3Δ1 leu2Δ0 met15Δ0 ura3Δ0 can1Δ::GAL1pr-SceI::STE2pr-SpHIS5 lyp1Δ::STE3pr-LEU2, NATIVEpr-sfGFP-PEX11*	Transformed with pMS894	α
YMS6012	sfGFP-Pxa1 *x* CFP-PTS1	*his3Δ1 leu2Δ0 met15Δ0 ura3Δ0 can1Δ::GAL1pr-SceI::STE2pr-SpHIS5 lyp1Δ::STE3pr-LEU2, NATIVEpr-sfGFP-PXA1*	Transformed with pMS894	α
YMS6013	sfGFP-Pex35 *x* CFP-PTS1	*his3Δ1 leu2Δ0 met15Δ0 ura3Δ0 can1Δ::GAL1pr-SceI::STE2pr-SpHIS5 lyp1Δ::STE3pr-LEU2, NATIVEpr-sfGFP-PEX35*	Transformed with pMS894	α
YMS6014	sfGFP-Pex25 *x* CFP-PTS1	*his3Δ1 leu2Δ0 met15Δ0 ura3Δ0 can1Δ::GAL1pr-SceI::STE2pr-SpHIS5 lyp1Δ::STE3pr-LEU2, NATIVEpr-sfGFP-PEX25*	Transformed with pMS894	α
YMS6015	sfGFP-Inp2 *x* CFP-PTS1	*his3Δ1 leu2Δ0 met15Δ0 ura3Δ0 can1Δ::GAL1pr-SceI::STE2pr-SpHIS5 lyp1Δ::STE3pr-LEU2, NATIVEpr-sfGFP-INP2*	Transformed with pMS894	α

YMS6016	sfGFP-Pex27 *x* CFP-PTS1	*his3Δ1 leu2Δ0 met15Δ0 ura3Δ0 can1Δ::GAL1pr-SceI::STE2pr-SpHIS5 lyp1Δ::STE3pr-LEU2, NATIVEpr-sfGFP-PEX27*	Transformed with pMS894	α
YMS6017	sfGFP-Pex19 x CFP-PTS1	*his3Δ1 leu2Δ0 met15Δ0 ura3Δ0 can1Δ::GAL1pr-SceI::STE2pr-SpHIS5 lyp1Δ::STE3pr-LEU2, NATIVEpr-sfGFP-PEX19*	Transformed with pMS894	α
YMS6018	sfGFP-Pex6 *x* CFP-PTS1	*his3Δ1 leu2Δ0 met15Δ0 ura3Δ0 lysx can1Δ::GAL1pr-SceI::STE2pr-SpHIS5 lyp1Δ::STE3pr-LEU2, NATIVEpr-sfGFP-PEX6*	Transformed with pMS894	α
YMS6019	sfGFP-Pex13 *x* CFP-PTS1	*his3Δ1 leu2Δ0 met15Δ0 ura3Δ0 can1Δ::GAL1pr-SceI::STE2pr-SpHIS5 lyp1Δ::STE3pr-LEU2, NATIVEpr-sfGFP-PEX13*	Transformed with pMS894	α
YMS6020	sfGFP-Pex21 x CFP-PTS1	*his3Δ1 leu2Δ0 met15Δ0 ura3Δ0 can1Δ::GAL1pr-SceI::STE2pr-SpHIS5 lyp1Δ::STE3pr-LEU2, NATIVEpr-sfGFP-PEX21*	Transformed with pMS894	α
YMS4473	*PEX11-MS2L*x Sec63-MCP-3xGFP	*his3Δ1 leu2Δ0 met15Δ0 ura3Δ0 PEX11::loxP::MS2L::PEX11_3'UTR,*	Transformed with pMS1248	α
YMS4472	*PEX14-MS2L*x Sec63-MCP 3xGFP	*his3Δ1 leu2Δ0 met15Δ0 ura3Δ0 PEX14::loxP::MS2L::PEX14_3'UTR,*	Transformed with pMS1248	α
YMS5938	*PEX14-MS2L* x Pnc1-mCherry	*his3Δ1 leu2Δ0 met15Δ0 ura3Δ0 PEX14::loxP::MS2L::PEX14_3'UTR, PNC1-mCherry-NAT*		α
YMS5938	*PEX14-MS2L* x MCP-GFP(x3) x Pnc1-mCherry	*his3Δ1 leu2Δ0 met15Δ0 ura3Δ0 PEX14::loxP::MS2L::PEX14_3'UTR, PNC1-mCherry::NAT*	Transformed with pMS196	α
YMS5938	*PEX14-MS2L* x Sec63-MCP(x2)-GFP x Pnc1-mCherry	*his3Δ1 leu2Δ0 met15Δ0 ura3Δ0 PEX14::loxP::MS2L::PEX14_3'UTR, PNC1-mCherry::NAT*	Transformed with pMS196 OR pMS1248	α
YMS5930	*PEX14-MS2L* x Om45-MCP(x2)-GFP	*his3Δ1 leu2Δ0 met15Δ0 ura3Δ0 PEX14::loxP::MS2L::PEX14_3'UTR, OM45-MCP(x2)-GFP::HIS*		α
YMS5322	*PXA1-MS2L*	*his3Δ1 leu2Δ0 met15Δ0 ura3Δ0 PXA1::loxP::MS2L::PXA1_3'UTR*	Described in (*22*).	α

YMS5933	*PXA1-MS2L x* Om45-MCP(x2)-GFP	*his3Δ1 leu2Δ0 met15Δ0 ura3Δ0 PXA1::loxP::MS2L::PXA1_3'UTR, OM45-MCP(x2)-GFP::HIS*		α
YMS6138	*Δpxa1*	*his3Δ1 leu2Δ0 met15Δ0 ura3Δ0 Δpxa1::G418*		α

**Table S3. List of plasmids used in this study**				
**Plasmid number**	**Plasmid name**	**Description**	**Plasmid use**	**Reference**
pMS894	pRS416_PEX5pr_CFP-SNAP-SKL LEU2	Yeast 2μ plasmid bearing *CFP-PTS1* (SKL) driven by *PEX5* promoter.	Visualize mature peroxisomes by cyan fluorescence color	Modified in this study to bear a LEU2 resistance cassette.
pMS618	pRS416_PEX5pr_CFP-SNAP-SKL URA3	Yeast 2μ plasmid bearing *CFP-PTS1* (SKL) driven by *PEX5* promoter.	Visualize mature peroxisomes in cyan fluorescence color	A kind gift from Prof. Ralf Erdmann.
pMS617	PRS315-mCherry-PTS1 LEU2	Yeast centromeric plasmid bearing mCherry cassette fused to PTS1	Visualize mature peroxisomes in red fluorescence color	A kind gift from Prof. Ralf Erdmann
pMS1006	PYM-Nop1pr-sfGFP-PEX14 URA3	PYM tagging plasmid bearing *NOP1pr-sfGFP-PEX14* for genomic integration.	Integrate *NOP1pr-sfGFP-PEX14* to HO locus	This study
pMS1007	PYM-noATG-sfGFP Nop1pr-PEX14 URA3	PYM tagging plasmid bearing *NOP1pr-sfGFP-PEX14* for genomic integration. The start methionine of the sfGFP was deleted.	Integrate *NOP1pr-noATG-sfGFP-PEX14* into the HO locus to visualize mRNA transcripts without protein translation.	This study
pMS11	pFA6a-KanMX6-PGAL1	PFA6a tagging plasmid used for tagging genes at their N' with *GAL*pr	For Pex19 expression under the *GALpr*	(51)
pMS506	pFA6a -mVenus-BirA::HIS5	PFA6a tagging plasmid to tag genes at their C' with mVenus-BirA	For tagging Pex25 and Pex11 with mVenus-BirA	
pMS278	PFA6a-mCherry-NAT	PFA6a tagging plasmid to tag genes at their C' with mCherry	Used to tag Pnc1 and Pex14 with mCherry at their C'.	

pMS1248	pRS416-SEC63-MCP(x2)- GFP URA3	Yeast 2μ expression plasmid bearing SEC*63-MCP-GFP(x2)* cassette.	Used as a tethering element.	Described in (*36*)
pMS196	MCP-GFPx3 HIS5	Yeast 2μ expression plasmid bearing MCP fused to 3 repeats of GFP.	Used to visualize mRNA *in vivo*	Described in (*22*).
pMS1104	PFA6-MCP(x2)-GFP- C' tagging HIS5	PFA6a tagging plasmid used for tagging a gene with two repeats of MCP fused to GFP.	Used to tether peroxisomal mRNA to the mitochondria.	This study

Movie S1. A movie with 3D representations of two peroxisomes and surrounding ribosomes that were imaged by corelative and light electron microscopy. Movie S1 is available in the online version of the manuscript.

REFERENCES AND NOTES

1. M. Veenhuis, J. M. Goodman, Peroxisomal assembly: Membrane proliferation precedes the induction of the abundant matrix proteins in the methyiotrophic yeast Candida boidinii. *J. Cell. Sci.* **6**, 583–590 (1990).

2. V. D. Antonenkov, J. K. Hiltunen, Transfer of metabolites across the peroxisomal membrane. *Biochim. Biophys. Acta* **1822**, 1374–1386 (2012).

3. R. Erdmann, W. Schliebs, Peroxisomal matrix protein import: The transient pore model. *Nat. Rev. Mol. Cell Biol.* **6**, 738–742 (2005).

4. Y. Fujiki, S. Nagata, Peroxisome biogenesis and human peroxisome-deficiency disorders. *Proc. Jpn. Acad. Ser. B Phys. Biol. Sci.* **92**, 463–477 (2016).

5. M. Islinger, A. Voelkl, H. D. Fahimi, M. Schrader, The peroxisome: An update on mysteries 2.0. *Histochem. Cell Biol.* **150**, 443–471 (2018).

6. F. D. Mast, R. A. Rachubinski, J. D. Aitchison, Peroxisome prognostications: Exploring the birth, life, and death of an organelle. *J. Cell Biol.* **219**, e201912100 (2020).

7. H. Rottensteiner, A. Kramer, S. Lorenzen, K. Stein, C. Landgraf, R. Volkmer-Engert, R. Erdmann, Peroxisomal membrane proteins contain common Pex19p-binding sites that are an integral part of their targeting signals. *Mol. Biol. Cell* **15**, 3406–3417 (2004).

8. W. Girzalski, L. S. Hoffman, A. Schemenewitz, A. Nolte, W. H. Kunau, R. Erdmann, Pex19p-dependent targeting of Pex17p, a peripheral component of the peroxisomal protein import machinery. *J. Biol. Chem.* **28**, 19417–19425 (2006).

9. W. B. Snyder, K. N. Faber, T. J. Wenzel, A.Koller, G. H. Luers, L. Rangell, G. A. Keller, S. Subramani, Pex19p interacts with Pex3p and Pex10p and is essential for peroxisome biogenesis in *Pichia pastoris*. *Mol. Biol. Cell* **10**, 1746–1761 (1999).

10. A. Halbach, R. Rucktäschel, H. Rottensteiner, R. Erdmann, The N-domain of Pex22p can functionally replace the Pex3p N-domain in targeting and peroxisome formation. *J. Biol. Chem.* **6**, 3906–3916 (2009).

11. R. L. M. Jansen, I. J. van der Klei, The peroxisome biogenesis factors Pex3 and Pex19: Multitasking proteins with disputed functions. *FEBS Lett.* **593**, 457–474 (2019).

12. B. A. Cichocki, K. Krumpe, D. G. Vitali, D. Rapaport, Pex19 is involved in importing dually targeted tail-anchored proteins to both mitochondria and peroxisomes. *Traffic* **19**, 770–785 (2018).

13. B. Schrul, W. Schliebs, Intracellular communication between lipid droplets and peroxisomes: The Janus face of PEX19. *Biol. Chem.* **399**, 741–749 (2018).

14. L. Wrobel, U. Topf, P. Bragoszewski, S. Wiese, M. E. Sztolsztener, S. Oeljeklaus, A. Varabyova, M. Lirski, P. Chroscicki, S. Mroczek, E. Januszewicz, A. Dziembowski, M. Koblowska, B. Warscheid, A. Chacinska, Mistargeted mitochondrial proteins activate a proteostatic response in the cytosol. *Nature* **524**, 485–488 (2015).

15. E. A. Costa, K. Subramanian, J. Nunnari, J. S. Weissman, Defining the physiological role of SRP in protein-targeting efficiency and specificity, *Science* **359**, 689–692 (2018).

16. O. Hermesh, R. P. Jansen, Take the (RN)A-train: Localization of mRNA to the endoplasmic reticulum. *Biochim. Biophys. Acta* **1833**, 2519–2525 (2013).

17. N. Aviram, M. Schuldiner, Targeting and translocation of proteins to the endoplasmic reticulum at a glance. *J. Cell Sci.* **130**, 4079–4085 (2017).

18. C. Lesnik, A. Golani-Armon, Y. Arava, Localized translation near the mitochondrial outer membrane: An update. *RNA Biol.* **12**, 801–809 (2015).

19. V. A. Gold, P. Chroscicki, P. Bragoszewski, A. Chacinska, Visualization of cytosolic ribosomes on the surface of mitochondria by electron cryo-tomography. *EMBO Rep.* **18**, 1786–1800 (2017).

20. R. Zoschke, R. Bock, Chloroplast translation: Structural and functional organization, operational control, and regulation. *Plant Cell* **30**, 745–770 (2018).

21. C. C. Williams, C. H. Jan, J. S. Weissman, Targeting and plasticity of mitochondrial proteins revealed by proximity-specific ribosome profiling. *Science* **346**, 748–751 (2014).

22. G. Zipor, L. Haim-Vilmovsky, R. Gelin-Licht, N. Gadir, C. Brocard, J. E. Gerst, Localization of mRNAs coding for peroxisomal proteins in the yeast, Saccharomyces cerevisiae. *Proc. Natl. Acad. Sci. U.S.A.* **106**, 19848–19853 (2009).

23. P. Lill, T. Hansen, D. Wendscheck, B. U. Klink, T. Jeziorek, D. Vismpas, J. Miehling, J. Bender, A. Schummer, F. Drepper, W. Girzalsky, B. Warscheid, R. Erdmann, C. Gatsogiannis, Towards the molecular architecture of the peroxisomal receptor docking complex. *Proc. Natl. Acad. Sci. U.S.A.* **117**, 33216–33224 (2021).

24. M. Komori, S. W. Rasmussen, J. A. K. W. Kiel, R. J. S. Baerends, J. M. Cregg, I. J. Van Der Klei, M. Veenhuis, The *Hansenula polymorpha PEX14* gene encodes a novel peroxisomal membrane protein essential for peroxisome biogenesis. *EMBO J.* **16**, 44–53 (1997).

25. E. Yifrach, S. Fischer, S. Oeljeklaus, M. Schuldiner, E. Zalckvar, B. Warscheid, Defining the mammalian peroxisomal proteome, in *Subcellular Biochemistry* (Springer, 2018), pp. 47–66.

26. K. Knoops, S. Manivannan, M. N. Cepińska, A. M. Krikken, A. M. Kram, M. Veenhuis, I. J. van der Klei, Preperoxisomal vesicles can form in the absence of Pex3. *J. Cell Biol.* **204**, 659–668 (2014).

27. A. Maekiniemi, R. H. Singer, E. Tutucci, Single molecule mRNA fluorescent in situ hybridization combined with immunofluorescence in *S. cerevisiae*: Dataset and quantification. *Data Brief* **30**, 105511 (2020).

28. F. Gallardo, P. Chartrand, Visualizing mRNAs in fixed and living yeast cells. *Methods Mol. Biol.* **714**, 203–219 (2011).

29. H. Wu, R. de Boer, A. M. Krikken, A. Akşit, W. Yuan, I. J. van der Klei, Peroxisome development in yeast is associated with the formation of Pex3-dependent peroxisome-vacuole contact sites. *Biochim. Biophys. Acta Mol. Cell. Res.* **1866**, 349–359 (2019).

30. K. Knoops, R. De Boer, A. Kram, I. J. Van Der Klei, Yeast pex1 cells contain peroxisomal ghosts that import matrix proteins upon reintroduction of Pex1. *J. Cell Biol.* **211**, 955–962 (2015).

31. W. Kukulski, M. Schorb, M. Kaksonen, J. A. G. Briggs, Plasma membrane reshaping during endocytosis is revealed by time-resolved electron tomography. *Cell* **150**, 508–520 (2012).

32. C. H. Jan, C. C. Williams, J. S. Weissman, Principles of ER cotranslational translocation revealed by proximity-specific ribosome profiling. *Science* **346**, 1257521 (2014).

33. N. Aviram, T. Ast, E. A. Costa, E. C. Arakel, S. G. Chuartzman, C. H. Jan, S. Haßdenteufel, J. Dudek, M. Jung, S. Schorr, R. Zimmermann, B. Schwappach, J. S. Weissman, M. Schuldiner, The SND proteins constitute an alternative targeting route to the endoplasmic reticulum. *Nature* **540**, 134–138 (2016).

34. R. S. Hamilton, I. Davis, Identifying and searching for conserved RNA localisation signals. *Methods Mol. Biol.* **714**, 447–466 (2011).

35. H. Rottensteiner, K. Stein, E. Sonnenhol, R. Erdmann, Conserved function of Pex11p and the novel Pex25p and Pex27p in peroxisome biogenesis. *Mol. Biol. Cell* **14**, 4316–4328 (2003).

36. D. Zabezhinsky, B. Slobodin, D. Rapaport, J. E. Gerst. An essential role for COPI in mRNA localization to mitochondria and mitochondrial function. *Cell Rep.* **15**, 540–549 (2016).

37. S. Gabay-Maskit, L. Daniel Cruz-Zaragoza, N. Shai, M. Eisenstein, C. Bibi, N. Cohen, T. Hansen, E. Yifrach, N. Harpaz, R. Belostotsky, W. Schliebs, M. Schuldiner, R. Erdmann, E. Zalckvar, A piggybacking mechanism enables peroxisomal localization of the glyoxylate cycle enzyme Mdh2 in yeast. *J. Cell Sci.* **133**, jcs244376 (2020).

38. A. M. Valm, S. Cohen, W. R. Legant, J. Melunis, U. Hershberg, E. Wait, A. R. Cohen, M. W. Davidson, E. Betzig, J. Lippincott-Schwartz, Applying systems-level spectral imaging and analysis to reveal the organelle interactome. *Nature* **546**, 162–167 (2017).

39. N. Shani, D. Valle, A Saccharomyces cerevisiae homolog of the human adrenoleukodystrophy transporter is a heterodimer of two half ATP-binding cassette transporters. *Proc. Natl. Acad. Sci. U.S.A.* **93**, 11901–11906 (1996).

40. N. Shani, P. A. Watkins, D. Vallett, J. W. Littlefield, J. Hopkins, PXA1, a possible Saccharomyces cerevisiae ortholog of the human adrenoleukodystrophy gene. *Proc. Natl. Acad. Sci. U.S.A.* **92**, 6012–6016 (1995).

41. M. Hanscho, D. E. Ruckerbauer, N. Chauhan, H. F. Hofbauer, S. Krahulec, B. Nidetzky, S. D. Kohlwein, J. Zanghellini, K. Natter, Nutritional requirements of the BY series of Saccharomyces cerevisiae strains for optimum growth. *FEMS Yeast Res.* **12**, 796–808 (2012).

42. R. D. Gietz, R. A. Woods, Transformation of yeast by lithium acetate/single-stranded carrier DNA/polyethylene glycol method. *Methods Enzymol.* **350**, 87–96 (2002).

43. I. Yofe, M. Schuldiner, Primers-4-Yeast: A comprehensive web tool for planning primers for *Saccharomyces cerevisiae*. *Yeast* **31**, 77–80 (2014).

44. A. Raj, P. van den Bogaard, S. A. Rifkin, A. van Oudenaarden, S. Tyagi, Imaging individual mRNA molecules using multiple singly labeled probes. *Nat. Methods* **5**, 877–879 (2008).

45. T. Lionnet, K. Czaplinski, X. Darzacq, Y. Shav-Tal, A. L. Wells, J. A. Chao, H. Y. Park, V. de Turris, M. Lopez-Jones, R. H. Singer, A transgenic mouse for in vivo detection of endogenous labeled mRNA. *Nat. Methods* **8**, 165–170 (2011).

46. J. Schindelin, I. Arganda-Carreras, E. Frise, V. Kaynig, M. Longair, T. Pietzsch, S. Preibisch, C. Rueden, S. Saalfeld, B. Schmid, J. Y. Tinevez, D. J. White, V. Hartenstein, K. Eliceiri, P. Tomancak, A. Cardona, Fiji: An open-source platform for biological-image analysis. *Nat. Methods* **9**, 676–682 (2012).

47. Y. S. Bykov, N. Cohen, N. Gabrielli, H. Manenschijn, S. Welsch, P. Chlanda, W. Kukulski, K. R. Patil, M. Schuldiner, J. A. G. Briggs, High-throughput ultrastructure screening using electron microscopy and fluorescent barcoding. *J. Cell Biol.* **218**, 2797–2811 (2019).

.48 J. R. Kremer, D. N. Mastronarde, J. R. McIntosh, Computer visualization of three-dimensional image data using IMOD. *J. Struct. Biol.* **116**, 71–76 (1996).

49. S. Berg, D. Kutra, T. Kroeger, C. N. Straehle, B. X. Kausler, C. Haubold, M. Schiegg, J. Ales, T. Beier, M. Rudy, K. Eren, J. I. Cervantes, B. Xu, F. Beuttenmueller, A. Wolny, C. Zhang, U. Koethe, F. A. Hamprecht, A. Kreshuk, ilastik: Interactive machine learning for (bio)image analysis. *Nat. Methods* **16**, 1226–1232 (2019).

50. U. Weill, I. Yofe, E. Sass, B. Stynen, D. Davidi, J. Natarajan, R. Ben-Menachem, Z. Avihou, O. Goldman, N. Harpaz, S. Chuartzman, K. Kniazev, B. Knoblach, J. Laborenz, F. Boos, J. Kowarzyk, S. Ben-Dor, E. Zalckvar, J. M. Herrmann, R. A. Rachubinski, O. Pines, D. Rapaport, S. W. Michnick, E. D. Levy, M. Schuldiner, Genome-wide SWAp-Tag yeast libraries for proteome exploration. *Nat. Methods* **15**, 617–622 (2018).

51. F. Madeira, Y. M. Park, J. Lee, N. Buso, T. Gur, N. Madhusoodanan, P. Basutkar, A. R. N. Tivey, S. C. Potter, R. D. Finn, R. Lopez, The EMBL-EBI search and sequence analysis tools APIs in 2019. *Nucleic Acids Res.* **47**, W636–W641 (2019).

52. U. Weill, N. Cohen, A. Fadel, S. Ben-Dor, M. Schuldiner, Protein topology prediction algorithms systematically investigated in the yeast *Saccharomyces cerevisiae*. *Bioessays* **41**, 1800252 (2019).
